# An O-RAN-Native xApp Framework for Jammer Detection and Geolocation

**DOI:** 10.3390/s26216859

**Published:** 2026-10-29

**Authors:** Steve Azarsh Ratti, Alex Piccioni, Fabio Graziosi, Dajana Cassioli, Andrea Marotta

**Affiliations:** DISIM Department, University of L’Aquila, 67100 L’Aquila, Italy; alex.piccioni@univaq.it (A.P.); fabio.graziosi@univaq.it (F.G.); dajana.cassioli@univaq.it (D.C.); andrea.marotta@univaq.it (A.M.)

**Keywords:** 5G security, jammer detection, jammer localization, Open Radio Access Network (O-RAN), physical-layer security, RAN Intelligent Controller (RIC), xApp

## Abstract

The Open Radio Access Network (O-RAN) architecture significantly enhances 5G networks by introducing modularity and intelligence through virtualization and open interfaces. While these networks provide advanced capabilities, the wireless medium remains inherently vulnerable to physical-layer (PHY) disruptions, with malicious radio frequency (RF) jamming posing a critical threat to network availability. To address this issue, this paper proposes a novel O-RAN-native architecture for real-time detection, identification, and localization of jamming attacks. Specifically, the proposed methodology leverages the coordination between specialized xApps running on the Near-Real-Time RAN Intelligent Controller (RIC) to perform real-time analysis of Key Performance Measurements (KPMs). The framework is evaluated in ns-O-RAN with a downlink jamming model, in which a wideband jammer of 40–60 dBm EIRP reduces the L3 SINR measured by the UEs, over 30 Monte Carlo realizations per configuration and a jammer-free control. The SINR-based stages detect the attack without false alarms in the jammer-free control runs and identify the affected sector. An interference-power trilateration that combines SINR and RSRP reports then localizes the jammer with a median error of 9–24 m for two jammer positions, four to ten times lower than centroid-based and path-loss trilateration baselines. The steady-state processing time of 0.89 s for 2000 UEs on a single core is within the 1 s control-loop budget of the Near-RT RIC when S-DBSCAN keeps one event per UE per second and the trilateration is initialized from the previous estimate. Note that these are processing times; the end-to-end latency including E2 transport was not measured. This work establishes a robust O-RAN-native framework for real-time PHY-layer jammer identification and geographic localization, thereby transforming standard network telemetry into actionable threat intelligence across multi-vendor deployments.

## 1. Introduction

Fifth-Generation (5G) and Beyond 5G (B5G) mobile networks mark a paradigm shift towards flexible, virtualized, and intelligent network architectures. While these advancements enable unprecedented connectivity and dynamic resource allocation, wireless networks remain inherently vulnerable to physical-layer (PHY) disruptions. Among these, malicious radio frequency (RF) jamming poses a critical and pervasive threat [1]. In a jamming attack, unauthorized devices emit high-power intentional interference to overpower legitimate signals, severely degrading network availability and perceived Quality of Service (QoS) for affected users. In traditional monolithic cellular architectures, detecting and mitigating such localized PHY threats is highly inflexible, typically requiring manual administrative intervention or dedicated external direction-finding hardware, both of which are operationally costly and incompatible with automated large-scale network management.

However, the Open Radio Access Network (O-RAN) paradigm offers a transformative platform for addressing these legacy physical-layer vulnerabilities. The O-RAN architecture disaggregates the traditional base station into distinct interoperable functional components, namely, the O-RAN Radio Unit (O-RU), O-RAN Distributed Unit (O-DU), and O-RAN Centralized Unit (O-CU). More importantly, it introduces the Near-Real-Time RAN Intelligent Controller (Near-RT RIC), a centralized abstraction layer that hosts specialized third-party software applications known as xApps. Through standardized open interfaces such as E2, xApps can continuously collect Key Performance Measurements (KPMs) from distributed base-station nodes. While xApps are frequently utilized to optimize general network performance such as load balancing [2] or intelligent traffic steering [3], their ability to ingest multi-node telemetry and execute Artificial Intelligence (AI)-driven control loops within 10 ms to 1 s makes them uniquely suited for advanced security monitoring [4].

By leveraging the programmable nature of the Near-RT RIC and its standard open interfaces, network operators can deploy machine learning-driven security xApps that continuously analyze network-wide data to manage resources and isolate anomalies [5,6]. While current research has extensively explored actionable mitigation, isolating an external interference source requires accurate geographic localization. Because O-RAN allows a Near-RT RIC to simultaneously aggregate signal degradation metrics from multiple adjacent User Equipment (UE) instances, it creates an unprecedented opportunity to detect attacks, identify the affected sector through multi-point spatial clustering, and localize the transmitter using the interference power inferred from the same reports, all without the need for dedicated hardware.

To address the critical security gap mentioned above, this paper demonstrates how the O-RAN architecture can be leveraged to proactively defend the physical layer by autonomously monitoring, detecting, and physically localizing unknown jamming devices. By transforming standard network telemetry into actionable threat intelligence, this approach ensures targeted network resilience against severe Denial-of-Service (DoS) disruptions. Specifically, this work makes the following contributions:

- An O-RAN-native architecture in which a KPM xApp and a Jammer xApp in the Near-RT RIC detect a jamming attack, identify the affected sector, and localize the jammer using only standard E2SM-KPM measurements without requiring any dedicated direction-finding hardware; long-term calibration can be delegated to an rApp in the Non-RT RIC.
- Two SINR-based detection stages based on the spatial clustering of the victim UEs: a stateful DBSCAN (S-DBSCAN), and a stateless hard-threshold HDBSCAN (HT-HDBSCAN).
- An interference power trilateration that estimates the jammer power each UE receives from its RSRP and L3 SINR reports and jointly fits the jammer position, EIRP, and path-loss exponent while requiring neither the jammer EIRP nor a pre-calibrated path loss model.
- An RIC-side UE positioning pipeline based on SINR-informed Maximum A Posteriori (SI-MAP) snapshot estimation and a causal Extended Kalman Filter (EKF) for deployments in which the UE positions are not reported, additionally evaluated under model mismatch.
- A downlink jamming extension of ns-O-RAN and a Monte Carlo evaluation (30 realizations per configuration, jammer-free control, separate training and test realizations) against centroid-based, minimum-SINR, and path loss trilateration baselines.

To validate the proposed framework, we model a downlink jamming attack in a standards-compliant O-RAN simulation platform (ns-O-RAN) in which a continuous wideband jammer adds interference to the SINR measured by the UEs. We evaluate the framework over multiple Monte Carlo realizations and against established baselines. Ultimately, bridging physical-layer RF metrics and O-RAN’s intelligent control plane [7] ensures targeted network resilience against critical security threats [8].

## 2. Related Work

O-RAN architectures require robust defense mechanisms, particularly real-time Intrusion Detection Systems (IDSs), to protect the physical layer [9,10]. As novel vulnerabilities emerge, current research has largely bifurcated into statistical thresholding and Machine Learning (ML) classification. On the statistical front, methodologies such as analyzing variations in noise variance have proven effective for immediate attack recognition [11]. Conversely, the ML domain has extensively used hybrid models [12], Random Forest, and Isolation Forest algorithms to detect complex throughput anomalies [13]. Furthermore, guided learning techniques such as Decision Trees and Support Vector Machines (SVMs) have demonstrated high efficacy on Key Performance Indicator (KPI) datasets [14]. At the edge of the RAN, ML-based xApps have proven capable of classifying malicious traffic [15], and low-resource edge AI solutions such as TinyML models on software-defined radios (SDRs) have successfully detected jamming in Internet of Things (IoT) environments [16]. To address “weak jamming” attacks that occur before full network disruption, recent work in [17] introduced an innovative approach that utilizes Convolutional Neural Networks (CNNs) and autoencoders to analyze raw In-phase and Quadrature (IQ) samples transformed into constellation images.

Recent surveys [18] have highlighted that security-focused xApps remain a critical research priority in O-RAN architectures. While some studies focus on internal or network-layer threats, such as “Rogue Cells” that manipulate telemetry to evade detection [19], other frameworks target broader DoS mitigation. For example, the system described in [20] integrates a distributed Application (dApp) for real-time monitoring with specialized xApps to analyze deviations in UE service consumption. To defend against adaptive jamming, frameworks such as Self-Adaptive Jammer Detection (SAJD) [21] employ self-adaptive retraining of models; in contrast, Federated Deep Reinforcement Learning (FDRL) systems [22] distribute agents across RICs to preserve privacy and mitigate attacks. Recent O-RAN-native research detects jamming by monitoring Block Error Rate (BLER) over the E2 interface. This mitigates the impact of jamming by capping the Modulation and Coding Scheme (MCS) on the gNB scheduler, with validation performed over-the-air on open-source Central Unit/Distributed Unit (CU/DU) platforms [23]. However, most current mitigation strategies remain reactive, such as relocating UE onto clear channels or stabilizing link parameters to counter interference [24], rather than neutralizing the physical source through geographic localization.

Despite these advances, a critical gap remains between passive anomaly detection and actionable geographic localization of external physical-layer threats. While 5G positioning techniques such as Millimeter-Wave (mmWave) and Multiple-Input Multiple-Output (MIMO) beamforming offer high accuracy [25], these capabilities are seldom integrated into standard O-RAN security xApps for real-time mitigation. Although initial steps toward O-RAN-native localization have demonstrated the feasibility of using power-based trilateration within the RIC to localize malicious UEs [26], such traditional methods may struggle in complex interference scenarios. Furthermore, the existing literature predominantly uses clustering algorithms such as Density-Based Spatial Clustering of Applications with Noise (DBSCAN) for logical traffic analysis. Such approaches group packet lengths or IP variability in order to identify Distributed Denial-of-Service (DDoS) and Address Resolution Protocol (ARP) attacks [27]. However, there is a notable scarcity of research that applies spatial clustering specifically to physical-layer security.

To bridge this gap, our study repurposes density-based clustering—specifically, DBSCAN [28] and Hierarchical Density-Based Spatial Clustering of Applications with Noise (HDBSCAN) [29]—for spatial anomaly detection. By correlating real-time Layer 3 Signal-to-Interference-plus-Noise Ratio (${\mathrm{SINR}}_{L3}$) degradation with the geographic (x,y) coordinates of victim UE, our framework demonstrates how spatial clustering can be applied in practice to physical-layer security, thereby helping to bridge the gap between theoretical models and real-world threat neutralization. The proposed methodology aligns with emerging adaptive jamming models [30]. As shown in Section 5, spatial clustering is effective for detecting the attack and identifying the affected sector. Localizing the transmitter additionally requires the interference power trilateration described in Section 4.3.3, which provides a practical enhancement to contemporary detection-only systems.

## 3. System Architecture

**Figure 1:**
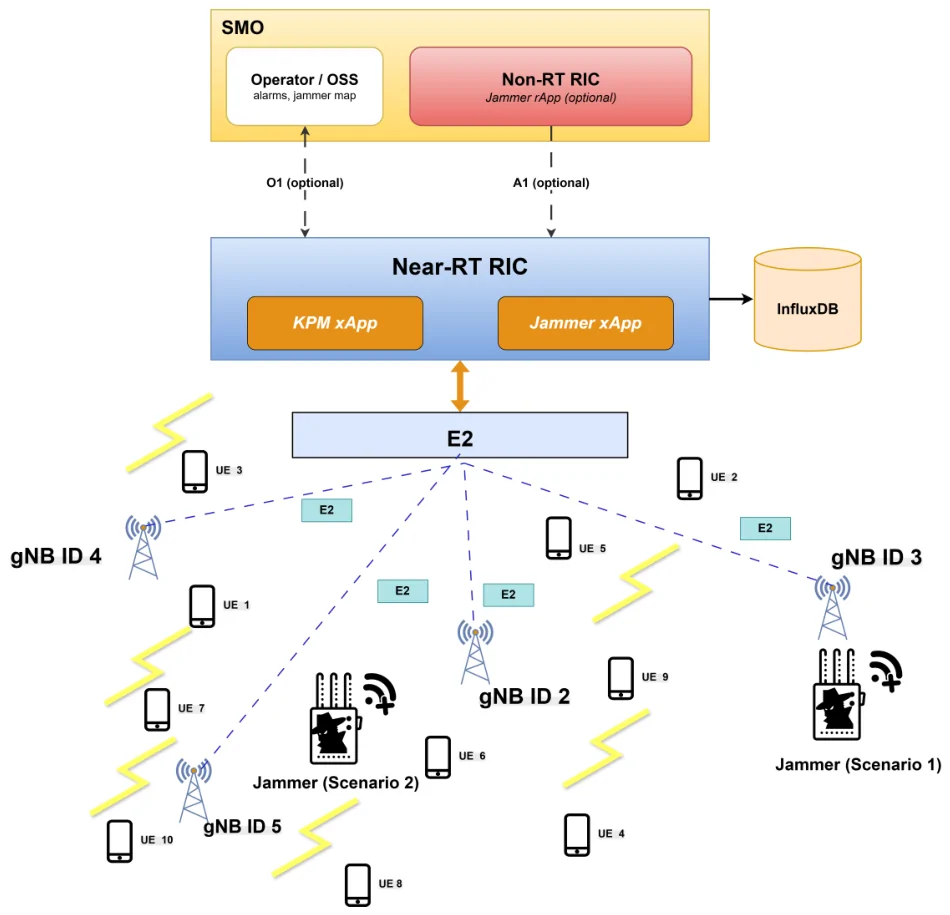
Deployment of the proposed framework. Four gNBs (E2 nodes) report KPMs through the E2 interface to the Near-RT RIC, which hosts the KPM xApp and the Jammer xApp; results are stored in InfluxDB. The Non-RT RIC resides in the SMO and can tune the Jammer xApp through A1; alarms and jammer positions are reported to the operator through O1 (both optional). The two jammers indicate the positions used in Scenario 1 (near gNB 3) and Scenario 2 (between gNB 5 and gNB 2); only one jammer is active in each simulation.

Figure 1 shows only the O-RAN elements that are relevant to this work. The gNBs act as E2 nodes and report Key Performance Measurements (KPMs) to the Near-RT RIC through the E2 interface, using the E2SM-KPM service model. These KPMs are the L3 SINR, the RSRP of the serving cell, the serving cell identifier, and when available, the UE position. The Near-RT RIC hosts the KPM xApp and the Jammer xApp, which perform the detection and localization described in Section 4. The Non-RT RIC, which resides in the Service Management and Orchestration (SMO) framework, is optional in our framework; it can host a Jammer rApp that calibrates the detection parameters over longer time scales and sends them to the Jammer xApp as A1 policies. Alarms and jammer positions are reported to the operator through the O1 interface. Other O-RAN interfaces, such as F1, E1, and the open fronthaul, are not involved in the proposed framework and are consequently omitted.

### 3.1. Architecture Overview and Data Flow

The telemetry and threat detection flow of Figure 1 operates as follows:

1. **Radio environment:** The gNBs serve the legitimate UEs in the downlink with useful signal power *S*. The jammer transmits continuous wideband interference with power *J* at each UE, so that the L3 SINR measured by the UE becomes S/(N+J), where *N* is the noise power.
2. **Measurement reporting:** The UEs report their measurements to the serving gNB, which collects the L3 SINR towards the serving and neighboring cells.
3. **Telemetry transport:** The gNBs forward these KPMs to the Near-RT RIC through the E2 interface (E2SM-KPM).
4. **Near-RT RIC processing:** The KPM xApp subscribes to KPMs and streams them to the Jammer xApp, which associates each SINR sample with the corresponding UE position. The position is either reported through E2 (Path A) or estimated in the RIC by the SI-MAP/EKF module of Section 4.2 (Path B).
5. **Detection, localization and alerting:** The Jammer xApp identifies victim UEs, declares the presence of a jammer, identifies the affected sector, and estimates the jammer position. The results are stored in a time series database (InfluxDB) and reported to the operator.

### 3.2. Core Components and Functions

**Figure 2:**
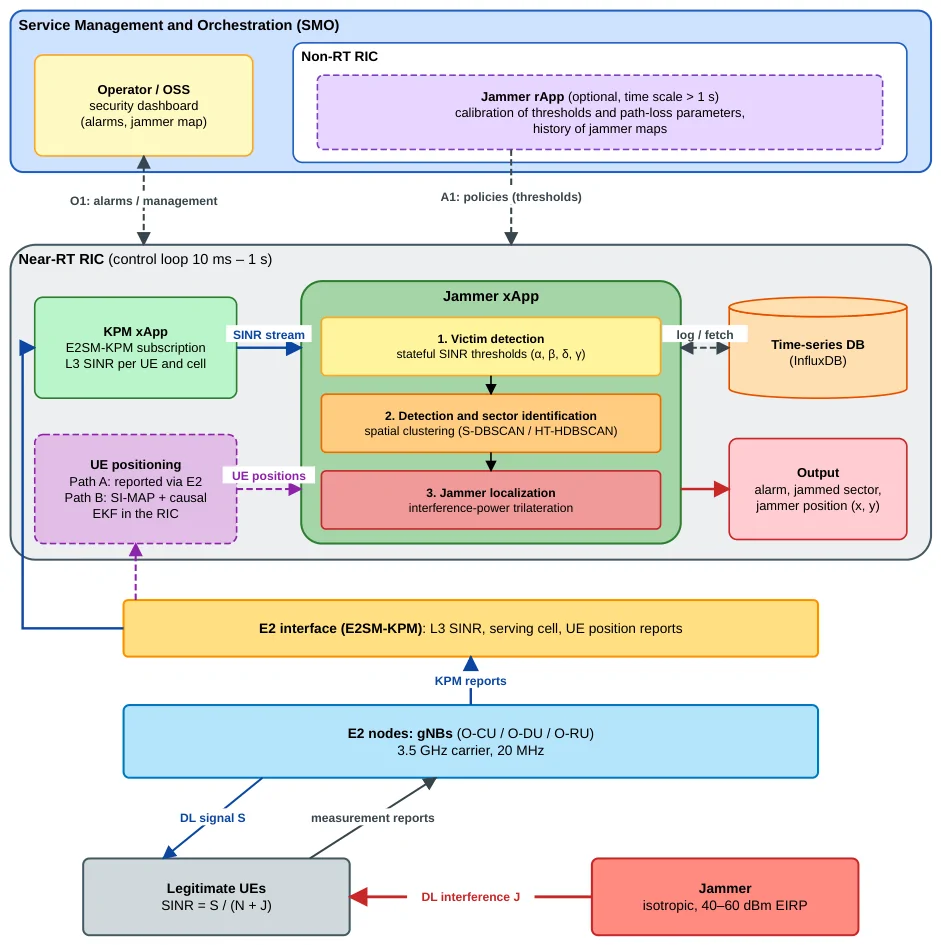
Logical architecture of the proposed framework. Solid elements are used in this work: E2 KPM telemetry from the gNBs feeds the KPM xApp and the Jammer xApp in the Near-RT RIC. Dashed elements are standard O-RAN components that are optional in our framework: the Non-RT RIC within the SMO can host a Jammer rApp that tunes the detection policies through A1, and alarms are reported to the operator through O1. The jammer acts on the downlink and reduces the L3 SINR measured by the UEs.

Figure 2 shows the framework’s logical architecture, from the radio environment through the gNBs and the E2 interface to the Near-RT RIC software modules. The main components are as follows:

- **Near-RT RIC:** Executes control loops between 10 ms and 1 s and hosts third-party applications (xApps); provides the RIC component, which can react to a jamming attack within about one second.
- **xApps:**
  – *KPM xApp:* Subscribes to the E2 nodes through E2SM-KPM and retrieves the layer 3 signal-to-interference-plus-noise ratio (${\mathrm{SINR}}_{L3}$) of every UE towards every cell. It corresponds to the telemetry component in Figure 2.
  – *Jammer xApp:* Associates the ${\mathrm{SINR}}_{L3}$ samples with the UE positions. Samples are processed in three stages: (i) victim detection with stateful SINR thresholds, (ii) detection of the attack and identification of the affected sector through spatial clustering (S-DBSCAN or HT-HDBSCAN), and (iii) localization of the jammer.
- **E2 interface:** Carries the KPM reports from the gNBs to the Near-RT RIC. It is the only O-RAN interface on which the proposed framework relies.
- **A1 and O1 interfaces (optional):** A1 can carry policies such as detection thresholds from a Jammer rApp in the Non-RT RIC to the Jammer xApp; O1 can carry alarms and jammer positions from the Near-RT RIC to the operator (Section 3.3).
- **Time series database (InfluxDB):** Stores the asynchronous streams of UE measurements and cell KPMs; aligns the UE positions and SINR samples in time.
- **Operator dashboard (OSS):** Displays the victim UEs, their serving cells, and the estimated jammer position on the network map. In a real deployment, the map can be provided by a Geographic Information System (GIS).
- **Jammer:** An external continuous wideband transmitter with an isotropic antenna and an equivalent isotropic radiated power (EIRP) between 40 and 60 dBm (10 W to 1 kW). It does not belong to the network and does not exchange any signaling with it; the framework localizes it only from the degradation of the ${\mathrm{SINR}}_{L3}$ observed by the legitimate UEs.

### 3.3. Deployment as dApp, xApp, or rApp

Industry and standardization activities have recently shifted attention from xApps towards the Non-RT RIC and rApps, while *dApps* have been proposed to host the real-time control loops that run below the Near-RT RIC [5]. Thus, the O-RAN application ecosystem spans three tiers with different time constraints: dApps at the O-DU/O-RU for real-time loops (below about 10 ms), xApps in the Near-RT RIC (10 ms–1 s), and rApps in the Non-RT RIC (above 1 s). The proposed framework maps naturally onto this ecosystem: Jammer detection and localization must process every KPM report (here, every 50 ms) and raise an alarm within about one second, which matches the Near-RT RIC control loop; therefore, it is deployed as an xApp and forms focus of this paper. A faster reactive response at the sub-10 ms scale, such as immediate mitigation at the O-DU/O-RU once a sector is flagged, would instead belong to a dApp, while the supporting long-term functions have no near-real-time constraint and can be implemented as rApps in the Non-RT RIC, which steer the detection xApp through A1 policies. These long-term functions include aggregation of jammer maps, calibration of thresholds and path loss parameters on historical data, and reporting to the operator. All of the results in Section 5 are obtained by offline processing of logged E2 KPM data, so the algorithms do not depend on the xApp runtime.

## 4. Methodology

The proposed framework operates in three stages: first, it continuously collects UE signal quality metrics through the xApp pipeline; second, it detects the attack and identifies the affected sector through spatial clustering; and third, it localizes the jamming source through interference power trilateration.

### 4.1. Monitoring Network Parameters

Radio environment monitoring relies on a KPM xApp operating in accordance with the O-RAN E2 Service Model for KPM [31]. The xApp continuously retrieves UE-specific performance parameters from the E2 nodes (gNBs) and logs them to a centralized time series database in order to establish a telemetry foundation for downstream detection and localization logic.

The aggregated telemetry serves critical operational functions; for instance, security-centric xApps leverage these metrics to detect anomalous interference, identify malicious jamming events, and prevent unauthorized network access. They also support spectrum monitoring functions, including received power density estimation and spectral occupancy analysis.

For signal quality monitoring, the KPM xApp retrieves the layer 3 signal-to-interference-plus-noise ratio (${\mathrm{SINR}}_{L3}$) individually for every active UE connected to each gNB. Specifically, ${\mathrm{SINR}}_{L3}$ quantifies the ratio of the received useful signal power to the combined interference and noise floor at the UE receiver: a high positive value (in dB) indicates a reliable radio link; in contrast, a low or negative value indicates that interference has overwhelmed the useful signal and severely degraded the connection. The Radio Resource Control (RRC) layer reports this per-UE metric; it sits above the raw physical layer and provides a stable averaged view of each device’s link quality. It is preferred over raw physical-layer snapshots because it directly captures whether a specific UE connection has become unusable, which is the signature of jamming-induced degradation. Once these per-UE ${\mathrm{SINR}}_{L3}$ readings are collected, the Jammer xApp associates each of them with the position of the UE at the time of the report. This position is either reported by the network or estimated in the RIC using the pipeline of Section 4.2. The result is a map of where the signal degradation occurs in space. Spatial clustering of severely affected UEs is then used to detect the attack and identify the affected sector. Finally, the interference power trilateration in Section 4.3.3 estimates the jammer coordinates without the need for specialized direction-finding hardware.

### 4.2. UE Position Estimation via Ranging and SI-MAP/EKF Tracking

Both the KPM monitoring pipeline and the jammer localization logic described below depend on an accurate estimate of each UE’s geographic position (x,y). Rather than assuming ideal error-free GPS reporting, we model UE position estimation as an explicit ranging-and-tracking problem. In a commercial 5G deployment, the xApp periodically collects a positioning snapshot for each UE, i.e., a single time instant at which range and angle observables from all visible gNBs are gathered. These observables are derived from network-assisted positioning signals such as the Positioning Reference Signal (PRS). Because they are inherently corrupted by thermal noise, multipath, and Non-Line-of-Sight (NLOS) propagation, the estimator has access only to these noisy observables, and never assumes that the true UE position is known. Since ns-O-RAN does not simulate the PRS, in our evaluation these observables are synthesized from the simulated geometry and the per-link SINR of the simulator according to the model below. The per-snapshot estimates are obtained with a SINR-Informed Maximum A Posteriori (SI-MAP) snapshot estimator, then refined by a causal Extended Kalman Filter (EKF). The remainder of this subsection develops this pipeline as four logically connected stages, each feeding the next: (1) the *Observable Model* specifies how the noisy per-snapshot range and angle observables are generated at each gNB; (2) the *SI-MAP Position Estimator* then converts these observables into a single position estimate for that snapshot; (3) the *Per-Snapshot Uncertainty (CRLB)* stage quantifies the confidence of that estimate as an anisotropic covariance; and (4) the *EKF Tracking* stage fuses the resulting sequence of snapshot estimates and their covariances over time into a filtered trajectory.

#### 4.2.1. Observable Model

For each UE and each serving/neighbor gNB *j*, a pseudo-range observation is modeled as

(1) $${\hat{\rho }}_{j}=d_{j}+b_{j}+n_{j},\;n_{j}∼N\left(0,\sigma_{j}^{2}\right),$$

where $d_{j}$ is the true Euclidean distance, $b_{j}$ is an NLOS excess delay bias, and $\sigma_{j}$ is the ranging standard deviation derived from the Cramér–Rao Lower Bound (CRLB) for time-of-arrival estimation:

(2) $$\sigma_{\tau ,j}=\frac{1}{2\pi \left(B/\sqrt{12}\right)\sqrt{2\cdot 10^{\gamma_{j}/10}}},\;\sigma_{j}=\max\left(\sigma_{\tau ,j}\cdot c,\;\sigma_{\min}\right),$$

with *B* as the channel bandwidth, $\gamma_{j}$ the per-link ${\mathrm{SINR}}_{L3}$ on the UE–gNB *j* link, *c* the speed of light, and $\sigma_{\min}$ a lower floor imposed on the ranging standard deviation to prevent numerically over-optimistic weights on high-${\mathrm{SINR}}_{L3}$ links. When a direct per-link ${\mathrm{SINR}}_{L3}$ measurement is unavailable, the link SINR $\gamma_{j}$ is obtained from the radio link budget as $P_{tx}-\mathrm{PL}-\left(I+N\right)$, where $P_{tx}$ denotes the gNB transmit power (in dBm), PL is the distance-dependent path loss (in dB), *I* represents the total co-channel interference power (in dBm), and *N* denotes the thermal noise floor power (in dBm). The Line-of-Sight (LOS)/NLOS state of each link evolves as a persistent (Gauss–Markov) process, with transition probability governed by the UE’s displacement between successive positioning snapshots; NLOS links receive an additional exponentially-distributed excess delay bias, as per the Third-Generation Partnership Project (3GPP) channel model. A complementary Angle-of-Arrival (AoA) observation ${\hat{\theta }}_{j}=\theta_{j}+n_{\theta ,j}$ is also modeled at each gNB, where $\theta_{j}$ is the true geometric angle of arrival and $n_{\theta ,j}$ is zero-mean Gaussian angular measurement noise. The variance of $n_{\theta ,j}$ is scaled inversely with the observed ${\mathrm{SINR}}_{L3}$, reflecting degraded angular resolution on weak links.

#### 4.2.2. SI-MAP Position Estimator

At each snapshot, the UE position is estimated as the point p=(x,y) that maximizes a soft LOS/NLOS mixture likelihood (SI-MAP) rather than assuming a single hard-switched channel state. For each anchor *j*, the posterior probability of a LOS condition is obtained by combining a geometric LOS prior $P_{\mathrm{LOS}}\left(d_{j}\right)$ with an ${\mathrm{SINR}}_{L3}$-residual likelihood that compares the observed ${\mathrm{SINR}}_{L3}$ against the expected path loss, where $L_{\mathrm{LOS},j}$ and $L_{\mathrm{NLOS},j}$ denote the likelihood functions for the LOS and NLOS conditions, respectively:

(3) $$p_{L,j}=\frac{P_{\mathrm{LOS}}\left(d_{j}\right)\;L_{\mathrm{LOS},j}}{P_{\mathrm{LOS}}\left(d_{j}\right)\;L_{\mathrm{LOS},j}+\left(1-P_{\mathrm{LOS}}\left(d_{j}\right)\right)L_{\mathrm{NLOS},j}}.$$

The position estimate is then obtained by minimizing the negative log-likelihood:

(4) $$\hat{p}=\arg\min_{p}\;-\sum_{j}\log\left[p_{L,j}\;N\left(r_{j};0,\sigma_{j}^{2}\right)+\left(1-p_{L,j}\right)\;f_{\mathrm{EMG}}\left(r_{j};\sigma_{j},\mu \right)\right]$$

where $r_{j}={\hat{\rho }}_{j}-∥p-a_{j}∥$ is the range residual to gNB *j* (at location $a_{j}$), N(·) denotes the Gaussian density, and $f_{\mathrm{EMG}}$ is an Exponentially Modified Gaussian (EMG) density that captures the one-sided and positively skewed excess delay characteristic of NLOS propagation, with μ the mean of its exponential (excess-delay) component. When AoA is enabled, a corresponding angular residual term is added to the objective; this is weighted by $1/\sigma_{\theta ,j}^{2}$, where $\sigma_{\theta ,j}$ is the angular (AoA) standard deviation on link *j*. As this optimization is non-convex, a global grid search followed by a local derivative-free refinement is employed to avoid convergence to local minima, with the numerical implementation detailed in Section 5.

#### 4.2.3. Per-Snapshot Uncertainty (CRLB)

For each snapshot, the Fisher information matrix is assembled from the range and (if enabled) AoA Jacobians across all anchors, then inverted to obtain the full anisotropic covariance $P_{\mathrm{CRLB}}$ of the position estimate. This covariance is not merely a diagnostic; it is propagated forward as the per-snapshot measurement covariance in the tracking stage below, meaning that snapshots obtained under poor anchor geometry or low ${\mathrm{SINR}}_{L3}$ are automatically downweighted.

#### 4.2.4. EKF Tracking

Raw per-snapshot estimates are refined using a constant-velocity EKF with state $x={\left[x,y,v_{x},v_{y}\right]}^{⊤}$ (where $v_{x},v_{y}$ are the velocity components), process noise scaled to the UE’s maximum expected speed, and measurement covariance $R_{k}=P_{\mathrm{CRLB},k}$ (floored to avoid overconfidence on rare near-singular geometries). The filter is initialized from the first snapshot. A chi-squared innovation gate (99%, two degrees of freedom) rejects outlier snapshots before the update step. If ten consecutive snapshots (1 s) are rejected, the track is considered lost and the filter is re-initialized at the median of these rejected snapshots, allowing it to recover after a long NLOS outage.

Because the EKF uses only the current and past snapshots, each position estimate is available as soon as its snapshot is processed, which is compatible with the near-real-time control loop; no non-causal smoothing is applied. The filtered position $\left(x_{\mathrm{est}},y_{\mathrm{est}}\right)$ can be forwarded to the Jammer xApp in place of an idealized GPS fix (Path B in Figure 2). In the jammer-localization experiments of Section 5, Path B is emulated by taking the ground-truth positions and adding a temporally correlated error of the same order of magnitude as the error of this tracker (Section 5.1); in this way, the two evaluations remain independent. To avoid evaluating the estimator only on observables generated by its own model, we also test it under model mismatch, where the observables are generated with a larger NLOS excess delay, larger ranging noise, lower LOS probability, and AoA bias, all of which are unknown to the estimator.

### 4.3. Jammer Detection and Localization Logic

Prior to classification, the system must synchronize two asynchronous data streams: ${\mathrm{SINR}}_{L3}$ logs reported by the xApp, and the UE positions reported over E2 or estimated as in Section 4.2. A key challenge is that network signal metrics and UE position estimates are recorded at different rates. To address this, we apply a time-aligned merge strategy: for each ${\mathrm{SINR}}_{L3}$ measurement at time $t_{\mathrm{sinr}}$, the most recent available UE position (x,y) at or before $t_{\mathrm{sinr}}$ is associated with that measurement. This ensures that every signal observation is tied to the estimated physical location of the UE at the moment of measurement, enabling accurate spatial analysis under realistic positioning uncertainty. Two spatial clustering variants are proposed: stateful (S-DBSCAN) and stateless (HT-HDBSCAN). Both are applied to every scenario with the same parameters. Their full mathematical formulation is presented in the subsections below.

#### 4.3.1. Stateful-DBSCAN (S-DBSCAN)

In S-DBSCAN, formalized in Algorithm 1, the system employs a two-phase mathematical framework comprising stateful anomaly tracking and DBSCAN with weighted centroid refinement. This approach groups the victim events into a dense spatial cluster and takes its weighted centroid as the jammer estimate, while the stateful hysteresis suppresses transient SINR drops.
**Algorithm 1:** Jammer Detection and Weighted Localization (S-DBSCAN)**Require:** 
Synchronized Dataset $D=\{\left(x_{r},y_{r},\gamma_{r},u_{r}\right)\}$ where $\left(x_{r},y_{r}\right)$ are the UE coordinates, reported or estimated (Section 4.2), Thresholds $\gamma_{\mathrm{detect}},\gamma_{\mathrm{severe}}$, Suspicion Limit $\delta_{\mathrm{score}}$, Penalties α,β, Clustering Params $\epsilon ,\mu_{pts}$, Outlier-Trim Percentile $\pi_{\mathrm{trim}}$**Ensure:** 
Estimated Jammer Location $L_{\mathrm{est}}=\left(x_{\mathrm{est}},y_{\mathrm{est}}\right)$1:**Stage 1: Stateful Victim Detection**2:Initialize score map $S\leftarrow \{u_{i}:0\;\forall \;u\in D\}$3:Initialize victim subset $P_{\mathrm{victims}}\leftarrow \emptyset$4:**for** each record r∈D **do**5:    **if** $\gamma_{r}<\gamma_{\mathrm{detect}}$ **then**6:        $S\left[u_{r}\right]\leftarrow S\left[u_{r}\right]+\alpha$7:    **else**8:        $S\left[u_{r}\right]\leftarrow \max\left(0,S\left[u_{r}\right]-\beta \right)$9:    **end if**10:    **if** $S\left[u_{r}\right]\geq \delta_{\mathrm{score}}$ **and** $\gamma_{r}<\gamma_{\mathrm{severe}}$ **then**11:        $P_{\mathrm{victims}}\leftarrow P_{\mathrm{victims}}\cup \left\{\left(x_{r},y_{r},\gamma_{r}\right)\right\}$12:    **end if**13:**end for**14:**Stage 2: Spatial Clustering & Localization**15:**if** $|P_{\mathrm{victims}}|\geq \mu_{pts}$ **then**16:    $\mathrm{Clusters}\leftarrow \mathrm{DBSCAN}(P_{\mathrm{victims}},\epsilon ,\mu_{pts})$17:    C←LargestCluster(Clusters)18:    *Compute Weights via Quadratic Inverse SINR*19:    $\gamma_{\max}\leftarrow \max_{p\in C}\left(\gamma_{p}\right)$20:    **for** each point p∈C **do**21:        $w_{p}\leftarrow {\left(\gamma_{\max}-\gamma_{p}+1\right)}^{2}$22:    **end for**23:    *Calculate Initial Weighted Centroid*24:    $x_{\mathrm{rough}}\leftarrow \frac{\sum (p_{x}\cdot w_{p})}{\sum w_{p}}$25:    $y_{\mathrm{rough}}\leftarrow \frac{\sum (p_{y}\cdot w_{p})}{\sum w_{p}}$26:    $L_{\mathrm{rough}}\leftarrow \left(x_{\mathrm{rough}},y_{\mathrm{rough}}\right)$27:    *Refinement (Outlier Trim)*28:    **for** each point p∈C **do**29:        $d_{p}\leftarrow {∥p-L_{\mathrm{rough}}∥}_{2}$30:    **end for**31:    Remove *p* from C where $d_{p}>{\mathrm{Percentile}}_{\pi_{\mathrm{trim}}}\left(d\right)$32:    Recalculate $L_{\mathrm{est}}=\left(x_{\mathrm{est}},y_{\mathrm{est}}\right)$ using refined C33:**else**34:    $L_{\mathrm{est}}\leftarrow \mathrm{Null}$35:**end if**36:**return** $L_{\mathrm{est}}$

Let the synchronized telemetry dataset be defined as D, where each record r∈D is a tuple $\left(x_{r},y_{r},\gamma_{r},u_{r}\right)$ representing the Euclidean coordinates, the measured ${\mathrm{SINR}}_{L3}$ (in dB), and the unique UE identifier, respectively.

To mitigate false positives caused by transient channel fading or shadowing, the system applies a stateful hysteresis function. Let $S_{t}\left[u\right]$ denote the suspicion score for UE *u* at discrete time *t*. Given a primary detection threshold $\gamma_{\mathrm{detect}}$, for which the deployment-specific value is reported in Section 5, the state updates dynamically according to

(5) $$S_{t}\left[u\right]=\left\{\begin{array}{cc} S_{t-1}\left[u\right]+\alpha & \mathrm{if}\;\gamma_{r}<\gamma_{\mathrm{detect}}\\ \max(0,S_{t-1}\left[u\right]-\beta ) & \mathrm{otherwise}, \end{array}\right.$$

where the penalty factor α is weighted heavily relative to the recovery factor β in order to ensure that the system reacts quickly (after three consecutive degraded reports) to sudden severe signal drops indicative of an attack. In contrast, the smaller β prevents premature clearance of the suspicion score during transient channel improvements. The operational values for α, β, and all remaining clustering hyperparameters depend on the deployment environment, and are detailed in Section 5. A UE is promoted to the confirmed-victim set $P_{\mathrm{victims}}$ when its suspicion score satisfies $S_{t}\left[u\right]\geq \delta_{\mathrm{score}}$ while simultaneously reporting an ${\mathrm{SINR}}_{L3}$ below the severe filter threshold $\gamma_{\mathrm{severe}}$; formally, $P_{\mathrm{victims}}=\left\{\left(x_{r},y_{r},\gamma_{r}\right):S_{t}\left[u_{r}\right]\geq \delta_{\mathrm{score}}\wedge \gamma_{r}<\gamma_{\mathrm{severe}}\right\}\subseteq D$, where $\delta_{\mathrm{score}}$ is the suspicion declaration limit.

The algorithm subsequently applies DBSCAN to the subset $P_{\mathrm{victims}}$. The spatial search radius ϵ and the minimum cluster density $\mu_{pts}$ are defined as tunable hyperparameters. Both are chosen to align with the geometric properties of the target deployment so that victim UEs sharing a cell coverage area fall within the search radius of a single cluster.

Let $C\subseteq P_{\mathrm{victims}}$ define the largest resultant spatial cluster. This effectively isolates the densest concentration of interference while mathematically discarding isolated reports as noise. To estimate the jammer’s origin $L_{\mathrm{est}}$, we define an inverse-proportional weight function $w_{p}$ for each coordinate point p∈C. Let $\gamma_{p}$ represent the measured ${\mathrm{SINR}}_{L3}$ at coordinate point *p*, and let $\gamma_{\max}=\max_{p\in C}\left(\gamma_{p}\right)$ represent the highest (least degraded) ${\mathrm{SINR}}_{L3}$ value within the cluster. The weight is calculated as follows:

(6) $$w_{p}={\left(\gamma_{\max}-\gamma_{p}+1\right)}^{2}\;\forall p\in C.$$

This quadratic weight function penalizes higher ${\mathrm{SINR}}_{L3}$ values, aggressively anchoring the mathematical centroid to the nodes experiencing the deepest signal collapse. The initial weighted centroid $L_{\mathrm{rough}}=\left(x_{\mathrm{rough}},y_{\mathrm{rough}}\right)$ is computed as

(7) $$x_{\mathrm{rough}}=\frac{\sum_{p\in C}p_{x}\cdot w_{p}}{\sum_{p\in C}w_{p}},\;y_{\mathrm{rough}}=\frac{\sum_{p\in C}p_{y}\cdot w_{p}}{\sum_{p\in C}w_{p}}.$$

Finally, a spatial refinement pass trims systemic outliers. Let $d_{p}={∥p-L_{\mathrm{rough}}∥}_{2}$ denote the Euclidean distance ($\ell_{2}$-norm) between each cluster point $p=(p_{x},p_{y})$ and the initial weighted centroid $L_{\mathrm{rough}}=\left(x_{\mathrm{rough}},y_{\mathrm{rough}}\right)$, defined as

(8) $$d_{p}=\sqrt{{\left(p_{x}-x_{\mathrm{rough}}\right)}^{2}+{\left(p_{y}-y_{\mathrm{rough}}\right)}^{2}}.$$

Points where $d_{p}$ exceeds the $\pi_{\mathrm{trim}}$-th percentile of all calculated distances are removed from C, where $\pi_{\mathrm{trim}}$ is a tunable outlier rejection threshold; its operational value is detailed in Section 5. The final estimated jammer coordinates $L_{\mathrm{est}}=\left(x_{\mathrm{est}},y_{\mathrm{est}}\right)$ are then obtained by recomputing the weighted centroid over this refined subset, yielding a physical-layer localization target.

#### 4.3.2. Hard-Threshold HDBSCAN (HT-HDBSCAN)

HT-HDBSCAN, detailed in Algorithm 2, instead utilizes HDBSCAN. Unlike partition-based methods such as K-means, HDBSCAN does not require the number of clusters to be specified a priori and is inherently robust against spatial outliers (i.e., noise). It is a non-parametric stateless approach that uses a single hard SINR threshold, which makes it simpler; however, as shown in Section 5.4, it is also less sensitive to weak jammers.
**Algorithm 2:** Jammer Localization via SINR Thresholding and Density Clustering (HT-HDBSCAN)**Require:** 
Merged Dataset D containing synchronized ${\mathrm{SINR}}_{L3}$ and UE (x,y) coordinates for each UE, reported or estimated (Section 4.2), SINR Threshold $\gamma_{th}$, HDBSCAN Params $m_{cluster},m_{samples}$**Ensure:** 
Estimated Jammer Location ${\hat{L}}_{J}$1:**Stage 1: Anomaly Detection (Victim Identification)**2:V←∅                                ▹ Set of victim coordinates3:**for** each (x,y,γ)∈D **do**4:    **if** $\gamma <\gamma_{th}$ **then**                   ▹ Check if ${\mathrm{SINR}}_{L3}$ is below severe threshold5:        V←V∪{(x,y)}6:        Mark UE as *Jamming Victim*7:    **else**8:        Mark UE as *Normal*9:    **end if**10:**end for**11:**Stage 2: Spatial Clustering (Localization)**12:**if** $|V|<m_{samples}$ **then**13:    **return** *∅*                             ▹ Insufficient data to cluster14:**end if**15:$Clusters\leftarrow \mathrm{HDBSCAN}(V,m_{cluster},m_{samples})$          ▹ Density-based clustering using MRD16:${\hat{L}}_{candidates}\leftarrow \emptyset$17:**for** each cluster $C_{k}\in Clusters$ **do**18:    $Center_{k}\leftarrow \left(\mathrm{median}\left(x_{C_{k}}\right),\mathrm{median}\left(y_{C_{k}}\right)\right)$19:    Add $Center_{k}$ to ${\hat{L}}_{candidates}$20:**end for**21:${\hat{L}}_{J}\leftarrow \mathrm{SelectBestCandidate}\left({\hat{L}}_{candidates}\right)$                ▹ Select largest/densest cluster22:**return** ${\hat{L}}_{J}$

The mathematical foundation of HDBSCAN relies on transforming the spatial space according to underlying density. To isolate the attack origin, the algorithm first computes the core distance $core_{k}\left(p\right)$ for each victim UE *p*, defined as the geographic distance to its *k*-th nearest neighbor. To penalize sparse regions and prevent isolated noise reports from falsely chaining clusters together, the spatial coordinates (x,y) of all detected victim events are evaluated using the Mutual Reachability Distance (MRD) between two victim UEs *a* and *b*:

(9) $$d_{\mathrm{mreach}-k}\left(a,b\right)=\max\left\{\;core_{k}\left(a\right),\;core_{k}\left(b\right),\;d\left(a,b\right)\;\right\}$$

where $core_{k}\left(\cdot \right)$ is the core distance defined above (the geographic distance from a UE to its *k*-th nearest neighbor) and $d\left(a,b\right)=∥\left(x_{a},y_{a}\right)-\left(x_{b},y_{b}\right){∥}_{2}$ is the standard Euclidean distance between UEs *a* and *b*. The neighbor evaluation parameter *k* is strictly mapped to the algorithm’s minimum samples parameter $m_{samples}$. By building a minimum spanning tree over this transformed density space and extracting the most stable clusters over varying density thresholds, HDBSCAN isolates the true region of severe interference from transient network anomalies.

Once the victim clusters are identified, the algorithm estimates the jammer’s coordinates. For each identified cluster, the centroid is calculated using the coordinate-wise median. This estimator is significantly more robust to intra-cluster outliers than the arithmetic mean, as it minimizes the sum of absolute deviations along each spatial axis:

(10) $${\hat{L}}_{candidate}=\left(\mathrm{median}\left(x_{cluster}\right),\mathrm{median}\left(y_{cluster}\right)\right).$$

In the event that HDBSCAN identifies multiple distinct interference clusters, the system applies a selection heuristic (SelectBestCandidate) to isolate the dominant threat. Let $C_{max}$ define the cluster exhibiting the highest cardinality of victim events, such that

(11) $$|C_{max}|=\max_{i}\left|C_{i}\right|.$$

The final estimated location of the jammer is ${\hat{L}}_{J}$, formally defined as the robust median centroid of this dominant cluster:

(12) $${\hat{L}}_{J}=\left(\mathrm{median}\left(x_{C_{max}}\right),\mathrm{median}\left(y_{C_{max}}\right)\right).$$

This final computed coordinate is then projected onto the operator dashboard to visualize the estimated jammer location relative to the ground-truth position (GT).

#### 4.3.3. Interference Power Trilateration

The clustering stages above rely only on the L3 SINR, which depends on both the jammer power and the strength of the serving signal. Thus, a UE at the cell edge could appear as a victim due to its distance from the jammer, while a UE close to its gNB could remain above the thresholds even near the jammer. To estimate the jammer coordinates, the Jammer xApp additionally uses the Reference Signal Received Power (RSRP) $S_{u}$, which is reported over E2SM-KPM together with the SINR. Since $\gamma_{u}=S_{u}/\left(N_{u}+J_{u}\right)$, the jammer power received by UE *u* is estimated as follows (in linear units): 

(13) $${\hat{J}}_{u}=\frac{S_{u}}{\gamma_{u}}-N_{u}$$

where the noise power $N_{u}$ is known from the bandwidth and the noise figure. In the evaluation, $S_{u}$ and $\gamma_{u}$ are affected by Gaussian measurement errors with standard deviations of 2 dB and 1 dB, respectively; the jammer power computed by the simulator is never used. For each UE, the reports are grouped in windows of duration *W*. For each window, the xApp computes the median of ${\hat{J}}_{u}$ and the mean position of the UE. A window is retained as a victim observation when its interference-to-noise ratio ${\hat{J}}_{u}/N_{u}$ exceeds ${\mathrm{INR}}_{th}$, and an alarm is raised when at least $N_{\min}$ distinct UEs provide victim observations. To ensure that the parameter fitting is not underdetermined, the estimator accumulates K≥4 valid temporal observations across these distinct UEs before computing the joint robust least squares fit. Let ${\left\{\left(p_{k},{\hat{J}}_{k}\right)\right\}}_{k=1}^{K}$ denote the retained observations, with ${\hat{J}}_{k}$ in dBm. A log-distance model describes the jammer:

(14) $${\hat{J}}_{k}=Q-10\;n\log_{10}\max\left(∥p_{k}-p_{J}∥,1\;m\right)+\varepsilon_{k}$$

where $p_{J}=\left(x_{J},y_{J}\right)$ is the jammer position, *Q* combines the unknown jammer EIRP and the reference path loss, *n* is the path loss exponent, and $\varepsilon_{k}$ accounts for shadowing and measurement errors. The four unknowns are estimated jointly by robust least squares:

(15) $$\left({\hat{p}}_{J},\hat{Q},\hat{n}\right)=\arg\min_{p_{J},Q,n}\sum_{k=1}^{K}\rho \;\left({\hat{J}}_{k}-Q+10\;n\log_{10}\max\left(∥p_{k}-p_{J}∥,1\;m\right)\right)$$

where ρ(·) is the soft-$\ell_{1}$ loss with a scale of 6 dB, which limits the influence of observations affected by deep shadowing. The exponent *n* is constrained to [1.5,5], while $p_{J}$ is constrained to the deployment area. Since the problem is non-convex, a 7×7 grid of initial positions is screened with a closed-form cost, and only the few most promising seeds are refined (a coarse-to-fine multistart); in continuous operation, the estimator can instead be initialized from the previous estimate (Section 5.5). Unlike path loss trilateration [26], this estimator does not require the jammer EIRP or a pre-calibrated path loss model because both are estimated from the data. The affected sector is the gNB closest to ${\hat{p}}_{J}$.

## 5. Simulation Results

**Figure 3:**
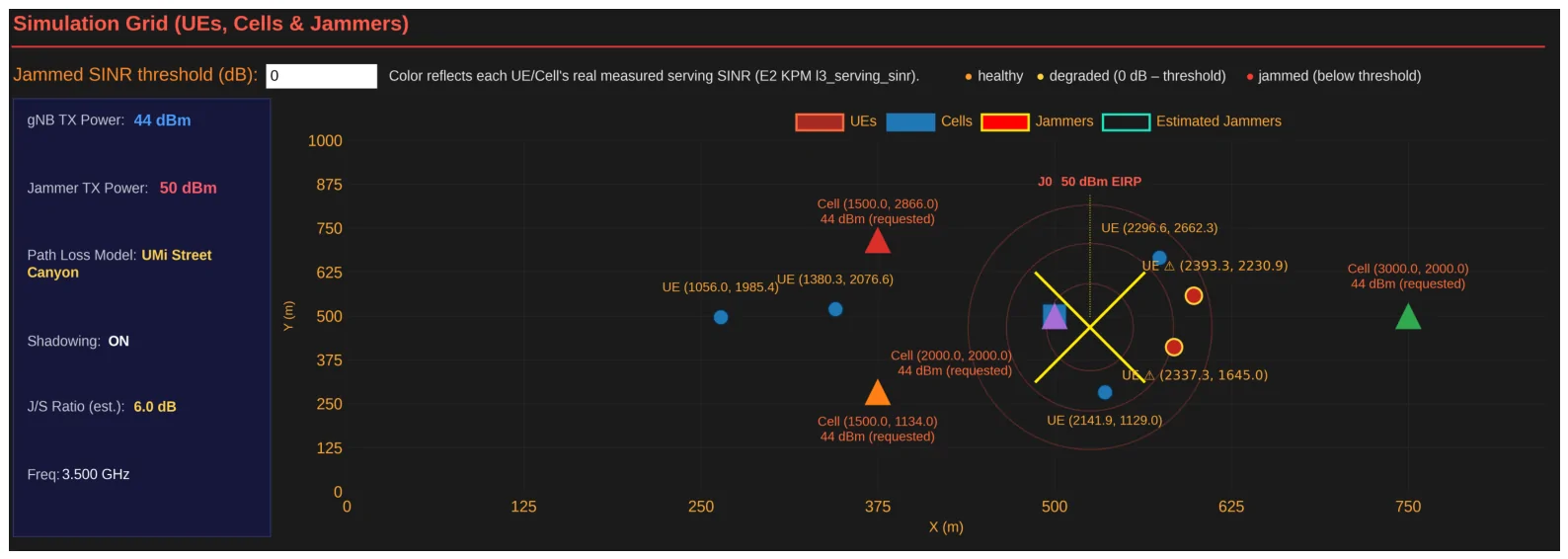
Real-time O-RAN jammer monitoring dashboard. The illustrative screenshot shows how the output of the Jammer xApp, consisting of the victim UEs, their serving cells, and the estimated jammer position, is presented to the operator in real time.

The proposed jamming detection and localization framework is implemented in Python 3.10.12, using the Pandas library (version 2.3.3) for time series manipulation, the scikit-learn library (version 1.7.2, including its HDBSCAN implementation) for spatial clustering, and SciPy (version 1.15.3) for the robust least squares fit of the interference power trilateration. The processing pipeline has three stages: data ingestion and synchronization, victim detection and clustering, and localization. The UE positioning pipeline of Section 4.2 is implemented separately in MATLAB R2025b. The SI-MAP position estimate is obtained by minimizing the negative log-likelihood of (4): the objective is first evaluated on a grid with 10 m spacing covering the whole deployment area, then the best grid point is refined with a Nelder–Mead simplex search. The operator-facing monitoring dashboard, shown in Figure 3, realizes the GUI/OSS block of the proposed architecture (detailed in Section 3.2). Rather than relying on a conceptual “simulation map” that would not exist in a physical deployment, this interface is designed as a real-time geographic visualization tool. In the dashboard, the main grid plots every UE and serving cell at its reported (x,y) position, with each marker shaded according to its live ${\mathrm{SINR}}_{L3}$—reported through the E2 KPM l3 serving sinr measurement—relative to an operator-adjustable threshold. In this way, nodes can be immediately distinguished as healthy, degraded, or jammed. To give operators a georeferenced view of the attack in real time, the gNBs are drawn as triangles and the jammer is drawn as a yellow cross surrounded by concentric range rings that indicate its interference range. The side panel surfaces the live radio configuration streamed over E2 (the gNB and jammer transmit powers, 3GPP UMi path loss model, carrier frequency, and estimated jamming-to-signal (J/S) ratio), providing immediate situational context alongside the spatial estimate. This interface is the point at which raw network telemetry is transformed into actionable threat intelligence.

To conduct the experimental evaluation and data collection, we utilized the ns-O-RAN framework [32,33], an open-source platform that integrates the Network Simulator 3 (ns-3) with FlexRIC, providing a highly configurable Near-RT RIC. This integrated environment enables the creation of a large-scale and standards-compliant 5G topology, providing the realistic E2 interface telemetry required to test our security xApps. Physical-layer jamming is modeled with a dedicated extension of the ns-O-RAN gNB PHY, as described below. While public O-RAN datasets (e.g., the Open Ireland testbed [34]) are invaluable for network-layer traffic classification, they currently lack the specific geographically-correlated ${\mathrm{SINR}}_{L3}$ degradation metrics required for localized physical-layer security.

Consequently, a custom simulation was needed in order to gather the dynamic location-aware KPMs required for validating our methodology. The simulator provides ground-truth UE coordinates (x,y), which are used for scoring and to emulate the case in which the network reports the UE positions (Path A in Figure 2). To assess the case in which the positions are estimated in the RIC (Path B), we also add to them a temporally correlated error (first-order Gauss–Markov, 10 s correlation time) with a standard deviation of 15 m per axis. This error is representative of the SI-MAP/EKF tracker of Section 4.2.

The use of this specific simulation environment is well established for this type of research, as prior work has successfully employed it to design and validate realistic RF jamming models against real-world measurements [35]. The channel is modeled using the 3GPP Urban Micro (UMi) channel model, as detailed in Table 7.4.1-1 of [36]. The simulation parameters defining the physical network scenario are detailed in Table 1. The jammer is modeled as an isotropic and continuous wideband transmitter located 1.5 m above ground, with EIRP $P_{J}\in \left\{40,50,60\right\}$ dBm (10 W to 1 kW), in line with commercially available high-power jammers. Its received power at UE *u* is $J_{u}=P_{J}-PL_{\mathrm{UMi}}\left(d_{J,u}\right)$, where $PL_{\mathrm{UMi}}$ is the 3GPP UMi Street Canyon path loss with LOS/NLOS state and shadowing at 3.5 GHz, computed independently of the gNB–UE channel. The L3 SINR reported over E2 is $\gamma_{u}=S_{u}/\left(N_{u}+J_{u}\right)$, with $N_{u}=-174+10\log_{10}B+NF$ dBm and NF=9 dB. Although the simulator module is named “mmWave”, the deployment operates at an FR1 carrier of 3.5 GHz with 20 MHz bandwidth, and gNBs and UEs use a single isotropic antenna element. Thus, the jammer affects the measured SINR; for this reason, the KPM reports and the handover decisions, but is not modeled at the transport-block decoding level. Inter-cell interference is neglected, as in the ns-O-RAN SINR report. Each configuration (two jammer positions, three jammer powers, and a jammer-free control) is simulated over 30 independent Monte Carlo realizations, which differ in UE mobility, shadowing, LOS/NLOS state and fading. The hyperparameters of the framework are summarized in Table 2. They were tuned on the first five realizations (seeds 1–5) only, and all reported results use the remaining realizations.

The evaluation considers two jammer positions (Table 1). In Scenario 1, the jammer is placed about 14 m from gNB 3 and mainly affects the UEs served by this cell. In Scenario 2, it is placed between gNB 5 and gNB 2 at about 100 m from both, so that the UEs of two cells are affected. Every detection and localization method is applied to both scenarios with the same parameters. Section 5.1 first evaluates the UE positioning pipeline; Section 5.2 and Section 5.3 illustrate the two scenarios on representative realizations, while Section 5.4 reports the aggregate results over all test realizations.

### 5.1. UE Position Estimation Performance

This subsection evaluates the UE positioning pipeline of Section 4.2 on the 30 jammer-free realizations of Scenario 1 (10 UEs, 60 s, one positioning snapshot every 100 ms). For the jammer-localization stages, the realizations with seeds 1–5 are used only for calibration: the interference-plus-noise level of the link budget used by SI-MAP (−70.2 dBm, one value per gNB) and the path-loss coefficient of the baseline are estimated on these five runs, then kept fixed. All of the results below are computed on the remaining 25 realizations (150,000 snapshots). As a baseline, we apply the path loss trilateration of [26] to the same snapshots: the per-link ${\mathrm{SINR}}_{L3}$ is converted into a distance with a single path-loss coefficient (η=2.90, calibrated on the training seeds), and the position is obtained by least squares trilateration.

**Figure 4:**
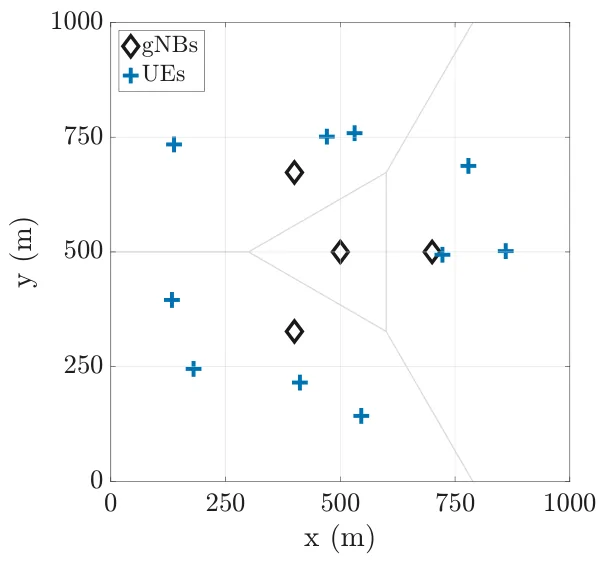
Simulated scenario geometry: gNB positions and initial UE positions (Scenario 1, seed 20). Grey lines: cell coverage boundaries.

**Figure 5:**
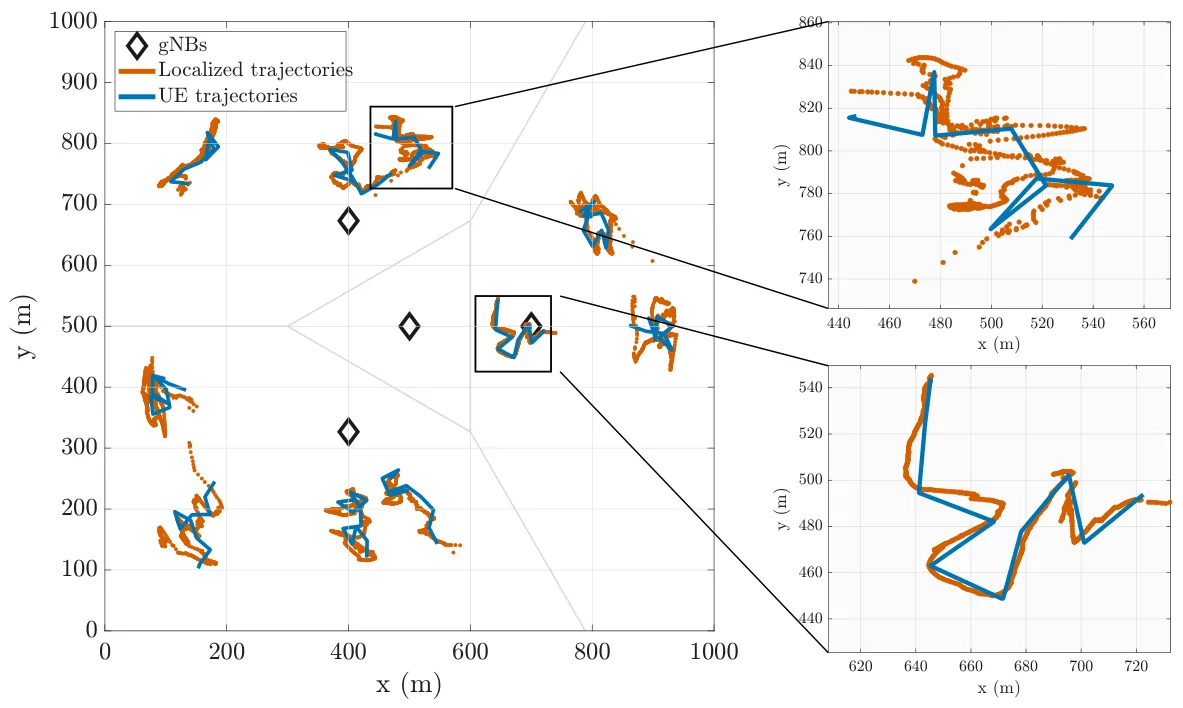
UE trajectory estimation with SI-MAP and the causal EKF (Scenario 1, seed 20, realization with the median error). The insets show UE 3 (**top**) and UE 7 (**bottom**). Grey lines: cell coverage boundaries.

Figure 4 shows the topology of the realization with the median tracking error (seed 20), and Figure 5 compares the true UE trajectories with the output of the causal EKF in the same realization. The estimated trajectories follow the true paths. The insets show UE 3, which moves outside the convex hull of the gNBs, and UE 7, which moves close to gNB 3. For UE 7, the estimate stays within a few meters of the true path; for UE 3, the EKF keeps the track, but errors of 10–20 m remain.

**Figure 6:**
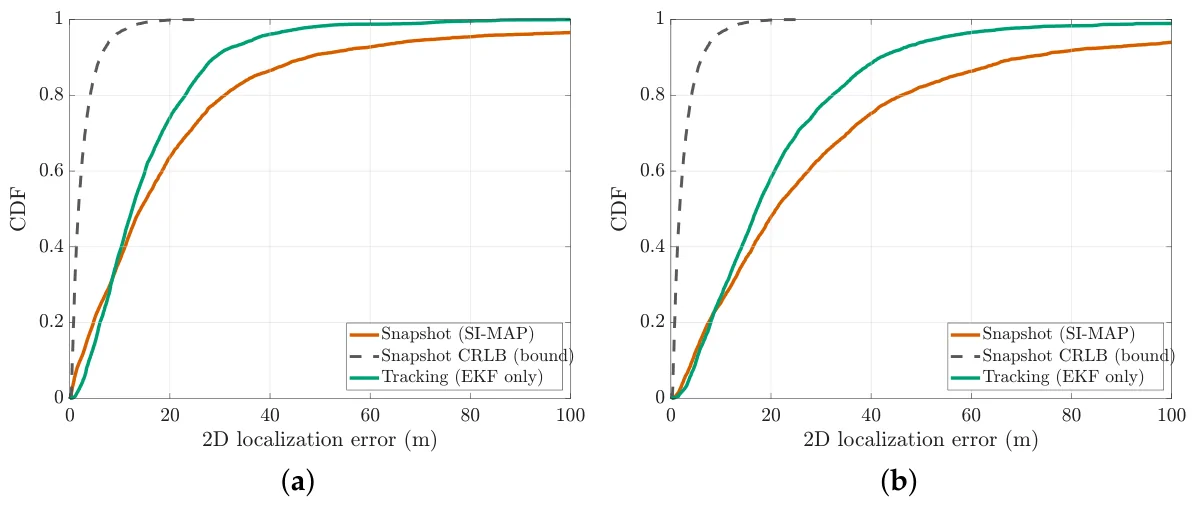
Empirical CDF of the 2D UE-localization error of the SI-MAP snapshot estimator and of the causal EKF, with the snapshot CRLB (Scenario 1, seed 20): (**a**) observables generated with the model assumed by the estimator and (**b**) observables generated with model mismatch.

Figure 6a shows the CDF of the 2D error in the same realization together with the CRLB. The CRLB (median 1.8 m) accounts only for the ranging and angle noise, not for the NLOS excess delay, which affects 93% of the links in this scenario. The gap between the CRLB and the SI-MAP error (median 14.2 m) is due mainly to the residual NLOS bias. The EKF reduces mostly the tail of the error; in this realization, the 90th percentile decreases from 47.4 m to 28.9 m.

Table 3 reports the errors pooled over all 25 test realizations. The causal EKF reduces the median error from 13.9 to 12.5 m, the 90th percentile from 53.4 to 33.5 m and the RMSE from 38.3 to 23.3 m. The median error of the EKF in each realization ranges between 8.7 and 16.0 m, so the result does not depend on a particular realization. The baseline [26] has a median error of 259 m. This is because a single path loss coefficient cannot account for the variation of the SINR due to shadowing, LOS/NLOS state and fading, and the resulting distance errors reach hundreds of meters.

To test the estimator when its observation model is not exact, the observables were also generated with a mean NLOS excess delay of 30 m instead of the 20 m assumed by SI-MAP, a ranging standard deviation 1.5 times larger than the CRLB-based value used by the estimator, a LOS probability equal to 0.7 times the 3GPP value, and a constant AoA bias of $2^{\circ }$. The estimator and its calibration were not changed. Under this mismatch, the median EKF error increases from 12.5 to 16.5 m and the RMSE from 23.3 to 34.4 m (Table 3 and Figure 6b), and the median error of each realization remains between 11.6 and 21.4 m. Therefore, the estimator degrades gradually when the model is not exact. The baseline is not affected because it uses only the SINR, which the mismatch does not modify. The same pipeline was applied without any change to the 30 jammer-free realizations of Scenario 2 (15 UEs), with the interference-plus-noise level and path loss coefficient of the baseline again calibrated on seeds 1–5 (−70.3 dBm and η=2.90). Over 25 test realizations (225,000 snapshots), the causal EKF attains a median error of 12.6 m, a 90th percentile of 33.7 m, and an RMSE of 23.6 m (Table 3), while the median error of each realization ranges between 9.0 and 16.4 m. These values are almost identical to those of Scenario 1. The accuracy is not determined by the number of UEs but by the gNB geometry and the NLOS conditions (87–95% of NLOS links), which are the same in both scenarios.

Under the 3GPP horizontal-error-at-80% metric (TS 22.261 / TR 38.855), the causal EKF reaches 24.3 m in Scenario 1 (32.4 m under model mismatch) and 24.2 m in Scenario 2, which does not meet the 10 m commercial outdoor target. In this scenario, the four gNBs are within about 350 m of each other, many UEs move outside their convex hull, 93% of the links are NLOS, and the bandwidth is 20 MHz; more anchors or a larger positioning bandwidth would be needed to meet the target. However, this accuracy is consistent with the UE position error used to emulate Path B in the jammer localization experiments; a Gauss–Markov error of 15 m per axis corresponds to a root mean square (RMS) 2D error of 21 m, close to the 23.3 m RMSE of the causal EKF. With this error, the median error of the interference power trilateration increases only from 9–24 m to 11–26 m (Section 5.4).

Per-UE errors for the realization of Figure 5 are reported in Table 4. The EKF reduces the mean absolute error of every UE along both axes, except along *x* for UE 2 (6.1 m instead of 6.0 m); on average, it decreases from 12.9 to 9.4 m along *x* and from 15.6 to 10.3 m along *y*.

In MATLAB, with no other simulation running on the computer (Scenario 2), SI-MAP takes 24 ms per UE snapshot on average with four gNBs, while EKF update takes 0.04 ms and the baseline takes 3.1 ms. When other simulations were running on the same computer (Scenario 1), these times increased to 127, 0.14, and 8.8 ms, respectively. The cost of SI-MAP is dominated by the grid search, which is independent for each UE and can be parallelized.

These results show that the UE positions required by Path B can be estimated in the RIC from network-side observables with a median error of 12.5 and 12.6 m in the two scenarios (16.5 m under model mismatch), which is the order of magnitude assumed in the jammer localization experiments.

### 5.2. Scenario 1: Localized Attack near gNB 3

**Figure 7:**
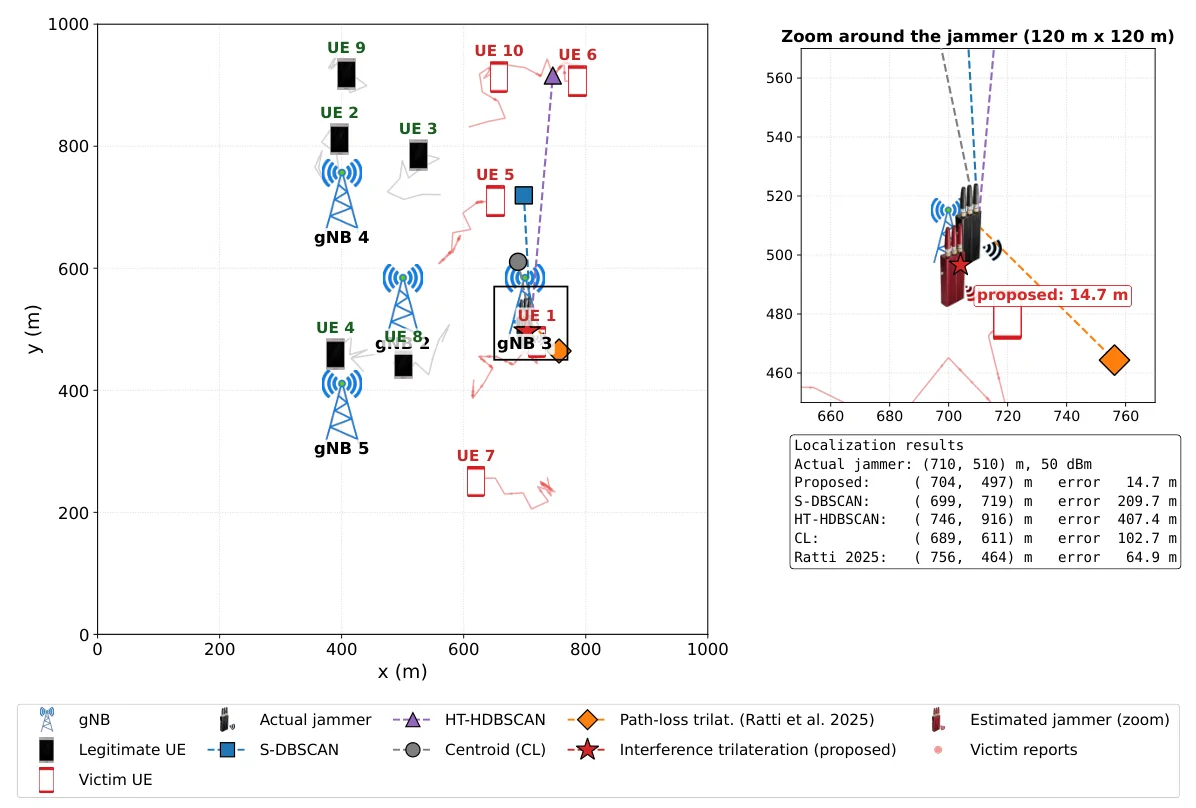
Jammer localization map for a representative realization of Scenario 1 (50 dBm jammer near gNB 3, test seed 24, median error of the proposed method). Towers: gNBs; red phones: victim UEs; black phones: legitimate UEs; thin lines: UE trajectories; black jammer: actual position. The markers show the estimates of S-DBSCAN, HT-HDBSCAN, centroid localization (CL), path loss trilateration [26], and the proposed interference power trilateration (star; red jammer in the zoom); the box lists the estimates and their errors.

In Scenario 1, the jammer is placed at (710,510) m, about 14 m from gNB 3, which models a worst-case attack against a single base station. The aggregate results over the 25 test realizations are reported in Section 5.4. Here, we illustrate the framework’s behavior on a representative realization (Figure 7), namely, the test run (seed 24) with a 50 dBm jammer, where the proposed method achieves its median localization error. The UE positions are those reported by the network (Path A).

**Figure 8:**
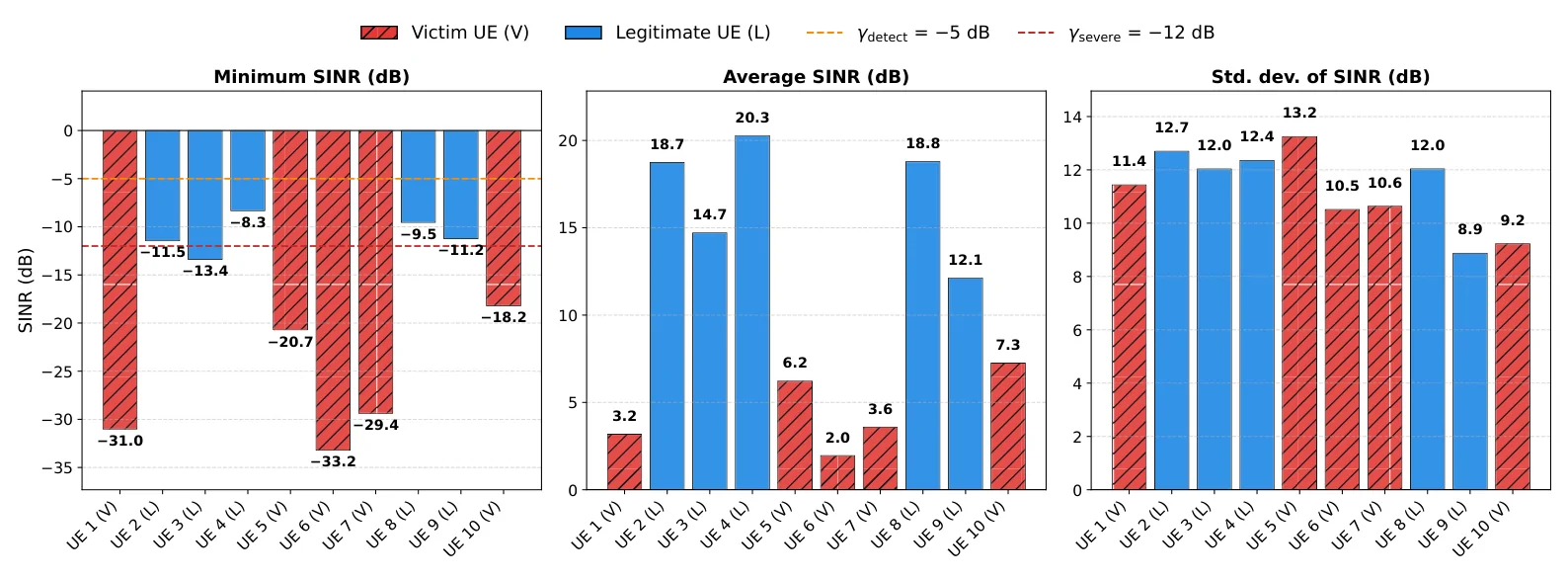
Minimum, average and standard deviation of the best-cell ${\mathrm{SINR}}_{L3}$ of each UE in the realization of Figure 7. Hatched red bars: victim UEs (V); blue bars: legitimate UEs (L); dashed lines: detection threshold $\gamma_{\mathrm{detect}}$ and severity threshold $\gamma_{\mathrm{severe}}$.

Victim identification follows Algorithm 1. Each ${\mathrm{SINR}}_{L3}$ report below $\gamma_{\mathrm{detect}}=-5$ dB increases the suspicion score of the UE by α, while each clean report decreases it by β. A UE is declared a victim when its score reaches $\delta_{\mathrm{score}}$ (i.e., after three consecutive degraded reports) and its ${\mathrm{SINR}}_{L3}$ is below $\gamma_{\mathrm{severe}}=-12$ dB. In this realization, five UEs were declared victims (UEs 1, 5, 6, 7, and 10) and five remained legitimate (Figure 8 and Table 5). The victims reached minimum SINR values between −33.2 and −18.2 dB and average values between 2.0 and 7.3 dB, whereas the legitimate UEs kept average values between 12.1 and 20.3 dB. The hysteresis suppressed short fades: UE 3 reached a minimum of −13.4 dB, but its degraded reports were too short-lived to satisfy both conditions of the stateful detector, so it was correctly kept as legitimate.

The victim set also illustrates the limitation of SINR-only localization discussed in Section 5.4. UEs 1 and 5 move within 200 m of the jammer, but UEs 6, 7, and 10 are also victims although they are 280–400 m away from it, far from every gNB, where the serving signal is weak; therefore the victim reports do not form a single cluster around the jammer. S-DBSCAN placed its estimate at (699,719) m, 210 m north of the jammer, HT-HDBSCAN at (746,916) m, 407 m away, and CL at (689,611) m, with an error of 103 m. Path loss trilateration [26], for which the nominal EIRP matches the true one in this realization, reached 65 m. Interference power trilateration estimated the jammer at (704,497) m, with an error of 14.7 m, and placed it in the sector of gNB 3 (Table 6).

**Figure 9:**
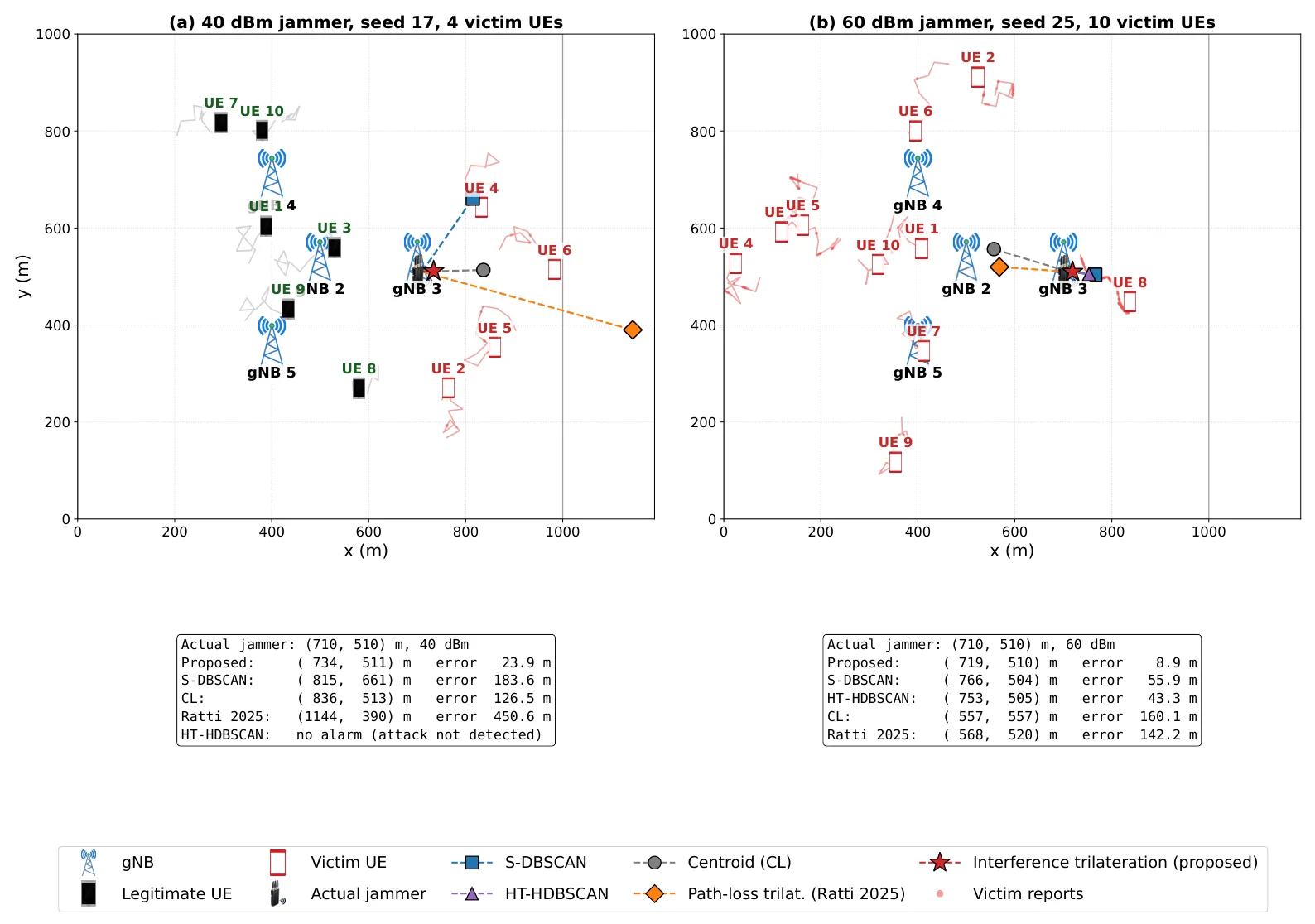
Median realizations of Scenario 1 at 40 dBm ((**a**), seed 17) and 60 dBm ((**b**), seed 25). The victim set grows from four UEs at 40 dBm to all ten UEs at 60 dBm, so estimates based on the victims’ positions are biased, whereas interference power trilateration attains errors of 23.9 and 8.9 m.

Figure 9 shows the median realizations at 40 and 60 dBm. The number of victims grows from four to ten UEs with the jammer power, and the errors of S-DBSCAN are 184 and 56 m; HT-HDBSCAN raises no alarm at 40 dBm and reaches 43 m at 60 dBm, while interference power trilateration attains 23.9 and 8.9 m.

### 5.3. Scenario 2: Jammer Between gNB 5 and gNB 2

**Figure 10:**
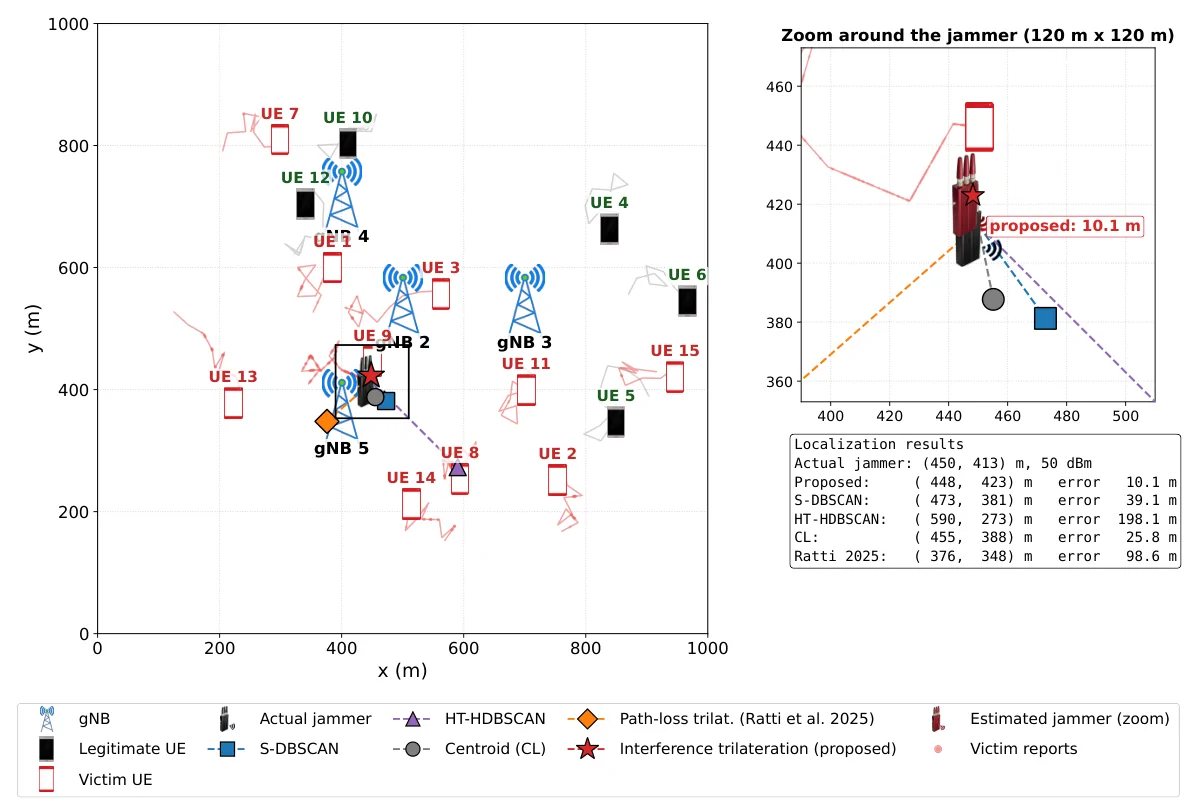
Jammer localization map for a representative realization of Scenario 2 (50 dBm jammer between gNB 5 and gNB 2, test seed 17, median error of the proposed method). Symbols as in Figure 7.

In Scenario 2, the jammer is placed at (450,413) m, at 99.6 m from gNB 5 and 100.3 m from gNB 2, meaning that its interference reaches the UEs of both cells. Since the two gNBs are almost equidistant from the jammer, an estimate is considered to be in the correct sector when the closest gNB is either gNB 5 or gNB 2. This scenario has fifteen UEs. All methods use the parameters tuned on the training seeds of Scenario 1, including the path loss coefficient of [26]; no parameter was tuned on Scenario 2. As in Scenario 1, we first illustrate a representative realization (Figure 10), namely, the test run with a 50 dBm jammer (seed 17), in which the proposed method attains its median localization error, with the UE positions reported by the network (Path A).

**Figure 11:**
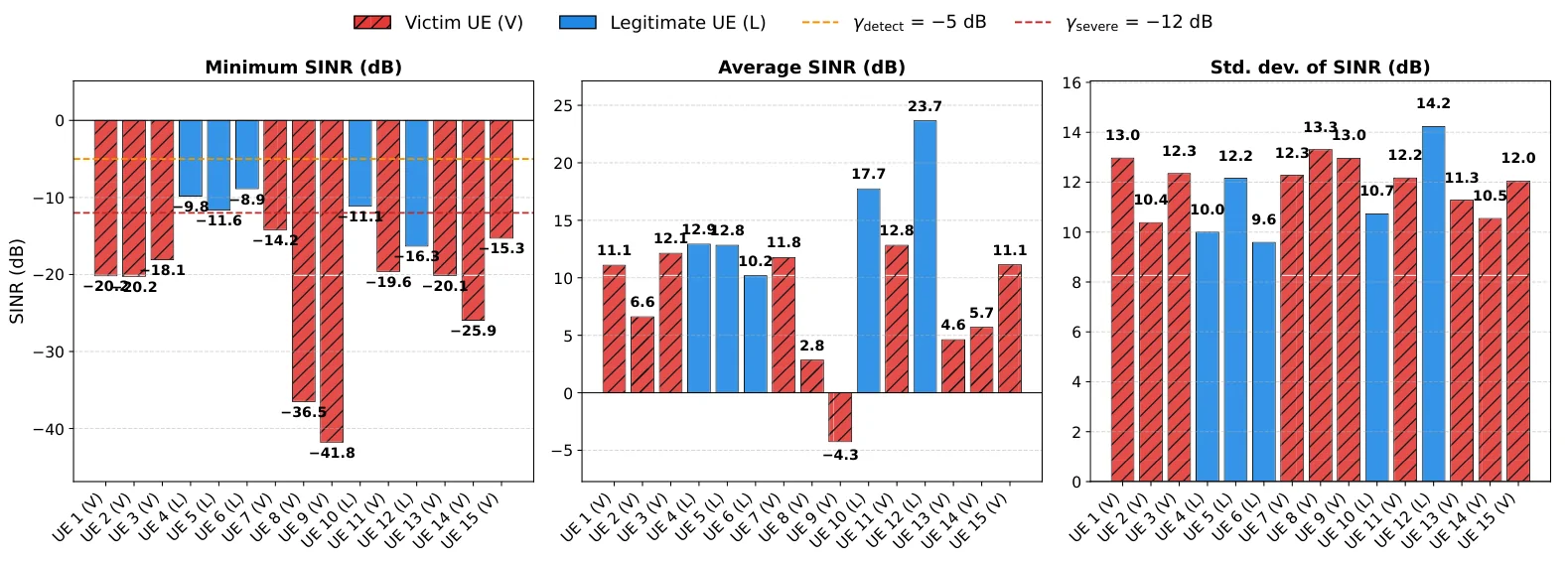
Minimum, average, and standard deviation of the best-cell ${\mathrm{SINR}}_{L3}$ of each UE in the realization of Figure 10. Hatched red bars: victim UEs (V); blue bars: legitimate UEs (L); dashed lines: detection threshold $\gamma_{\mathrm{detect}}$ and severity threshold $\gamma_{\mathrm{severe}}$.

In this realization, the stateful detector of Algorithm 1 declared ten UEs as victims (UEs 1, 2, 3, 7, 8, 9, 11, 13, 14, and 15) and five as legitimate (UEs 4, 5, 6, 10, and 12), as shown in Figure 11 and Table 7. The victims reached minimum SINR values between −41.8 and −14.2 dB and average values between −4.3 and 12.8 dB, whereas the legitimate UEs kept minimum values between −16.3 and −8.9 dB and average values between 10.2 and 23.7 dB. As in Scenario 1, the hysteresis kept UE 12 legitimate, though its SINR fell once to −16.3 dB. Unlike in Scenario 1, the victims are located on both sides of the boundary between the two cells.

Interference power trilateration estimated the jammer at (448,423) m, with an error of 10.1 m. The closest gNB is gNB 2 at 92.8 m, followed by gNB 5 at 107.6 m (Table 8), which is consistent with the true distances of 100.3 and 99.6 m. In this realization, CL (25.8 m) and S-DBSCAN (39.1 m) were also close to the jammer, as the victims surround it almost symmetrically; however, over all test realizations their median errors are 72 and 133 m (Section 5.4). Path loss trilateration [26] reached 98.6 m, and HT-HDBSCAN placed its estimate at (590,273) m, 198 m from the jammer, close to the trajectory of UE 8.

**Figure 12:**
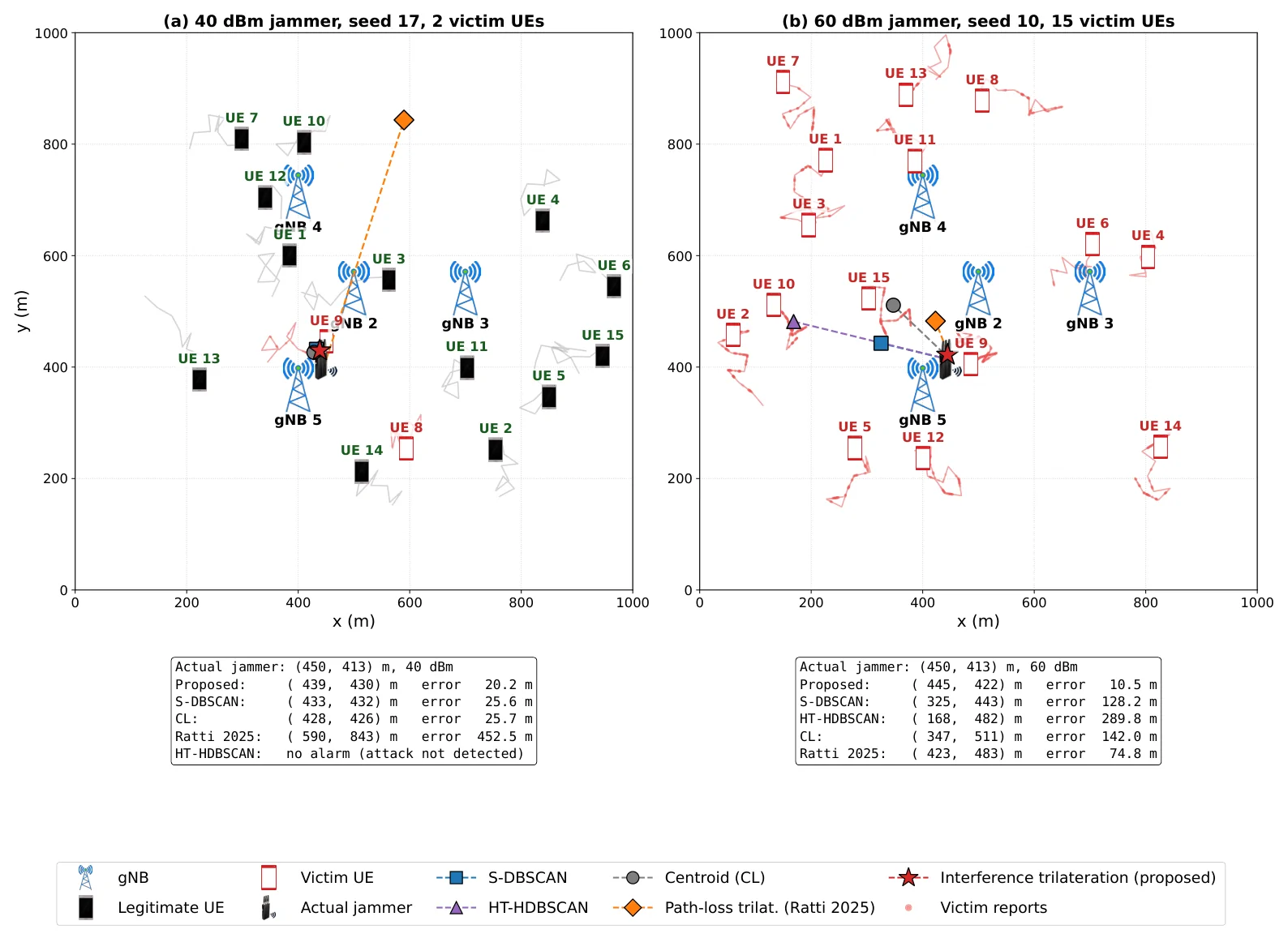
Median realizations of Scenario 2 at 40 dBm ((**a**), seed 17) and 60 dBm ((**b**), seed 10). The victim set grows from two UEs at 40 dBm to all fifteen UEs at 60 dBm, whereas interference power trilateration attains errors of 20.2 and 10.5 m.

Figure 12 shows the median realizations at 40 and 60 dBm. At 40 dBm, only UEs 8 and 9, which pass close to the jammer, become victims. In this realization, S-DBSCAN and CL reach 25.6 and 25.7 m, and HT-HDBSCAN raises no alarm. Path loss trilateration [26] places the jammer 453 m away; its nominal EIRP of 50 dBm is 10 dB higher than the true one, so it overestimates the distances between the jammer and the UEs. At 60 dBm, all fifteen UEs become victims, and the estimates based on the victim positions move towards the center of the victim set: S-DBSCAN, CL, and HT-HDBSCAN reach 128, 142, and 290 m, respectively, while path loss trilateration reaches 75 m. By comparison, interference power trilateration attains 20.2 and 10.5 m.

### 5.4. Comparison with Baseline Methods

To assess the proposed localization stages against established alternatives, every method was applied to every scenario with the same parameters and the same victim definition. The following methods were compared:

- **Centroid localization (CL):** The jammer position is the centroid of the positions of all victim reports, as in classic centroid-based jammer localization [37].
- **Minimum-SINR:** The jammer position is the position of the single most degraded report.
- **Path loss trilateration [26]:** Our earlier O-RAN localization method, applied to the jammer; this converts the interference power observed by each victim UE into a distance using the path loss model of [26], assuming a nominal jammer EIRP of 50 dBm, and solves a least squares trilateration.
- **S-DBSCAN and HT-HDBSCAN:** The clustering stages of Section 4.
- **Interference power trilateration (proposed):** The jammer power received by each UE is estimated as ${\hat{J}}_{u}=S_{u}/\gamma_{u}-N_{u}$ from the RSRP $S_{u}$ and the L3 SINR $\gamma_{u}$ reported over E2, then the jammer position, its EIRP, and the path loss exponent are fitted jointly using a robust least squares estimator.

**Figure 13:**
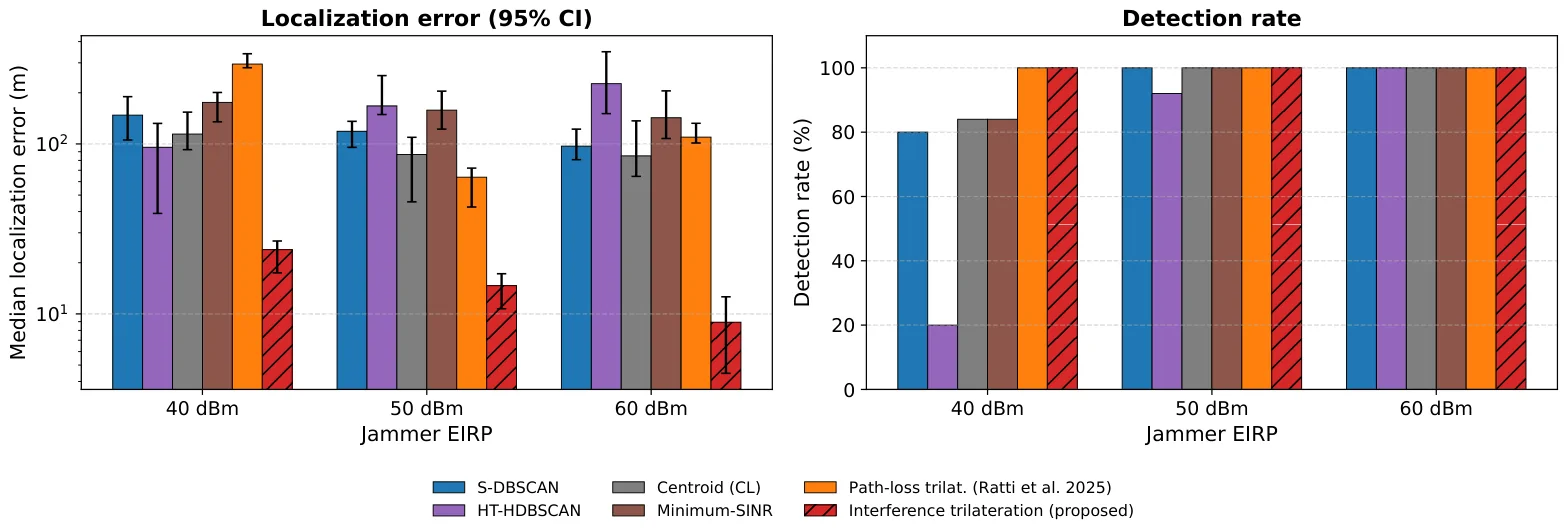
Scenario 1, 25 test realizations per jammer power: (**left**) median localization error of each method with its 95% bootstrap confidence interval (logarithmic scale); (**right**) detection rate.

Table 9 and Figure 13 report the results on the test seeds; seeds 1–5 were used only to tune the parameters, including the path loss coefficient of [26]. Detection and false alarm rates are computed over runs and over 1-s windows. The localization error is the median over the detected runs with its 95% bootstrap confidence interval.

No false alarms were observed in the jammer-free runs for any method (0 of 25 runs and 0 of 1500 one-second windows). With 25 independent runs, this is consistent with a false alarm probability below about 12% at the 95% confidence level; the one-second windows within a run are not independent, so they are reported only as an indication. The detection rate at 40 dBm, sector identification, and localization accuracy all differ substantially. Methods based only on the L3 SINR (CL, minimum-SINR, S-DBSCAN, and HT-HDBSCAN) obtain median errors between 96 and 175 m at 40 dBm and between 85 and 226 m at 60 dBm. Their error decreases only slightly as the jammer becomes stronger, and increases for HT-HDBSCAN. The reason is that the L3 SINR $S_{u}/\left(N_{u}+J_{u}\right)$ does not separate a strong jammer from a weak serving signal: UEs at the cell edge appear as victims even when far from the jammer, while UEs close to a gNB are protected by a strong serving signal even when near the jammer. Therefore, the victims form a ring around the jammer, which widens with the jammer power and biases the estimate. Moreover, because each UE reports every 50 ms, the victim events clustered by S-DBSCAN and HT-HDBSCAN are repeated samples of a few UEs, so their density also reflects how long each UE remains in the degraded region; interference power trilateration avoids this effect because it uses one observation per UE and per 1-s window, and requires at least $N_{\min}$ distinct UEs. The stricter threshold of HT-HDBSCAN reduces this effect at 40 dBm, but detects only 20% of the weak jammer runs. Among the SINR-only methods, S-DBSCAN identifies the correct sector most reliably (97% of the detected runs), but detects only 80% of the attacks at 40 dBm. Interference power trilateration removes the ambiguity by estimating the received jammer power from the RSRP and the SINR, both of which are standard E2SM-KPM measurements. It detects every attack, identifies the correct sector in all runs, and reduces the median error to 24, 15, and 9 m at 40, 50, and 60 dBm, respectively, i.e., four to ten times lower than the best alternative method at each power. With a correlated UE positioning error of 15 m per axis, which is representative of the SI-MAP/EKF tracker, these median errors become 26, 16, and 12 m, respectively, preserving the gain over the other methods. Path loss trilateration [26] with its path loss coefficient calibrated on the training seeds for the jammer localization task (η=2.92; the value η=2.90 of Section 5.1 refers to the separate UE positioning calibration) and a nominal jammer EIRP of 50 dBm reaches 64 m when the nominal EIRP matches the true one (50 dBm); its error grows to 110 m at 60 dBm and 295 m at 40 dBm, since the jammer EIRP is unknown to the defender. Even when the true EIRP is provided, its error remains 48–198 m, which is because a single path loss model with fixed coefficients cannot fit both nearby and distant UEs. The proposed method avoids both of these limitations by estimating the jammer EIRP and the path loss exponent jointly with the position.

**Figure 14:**
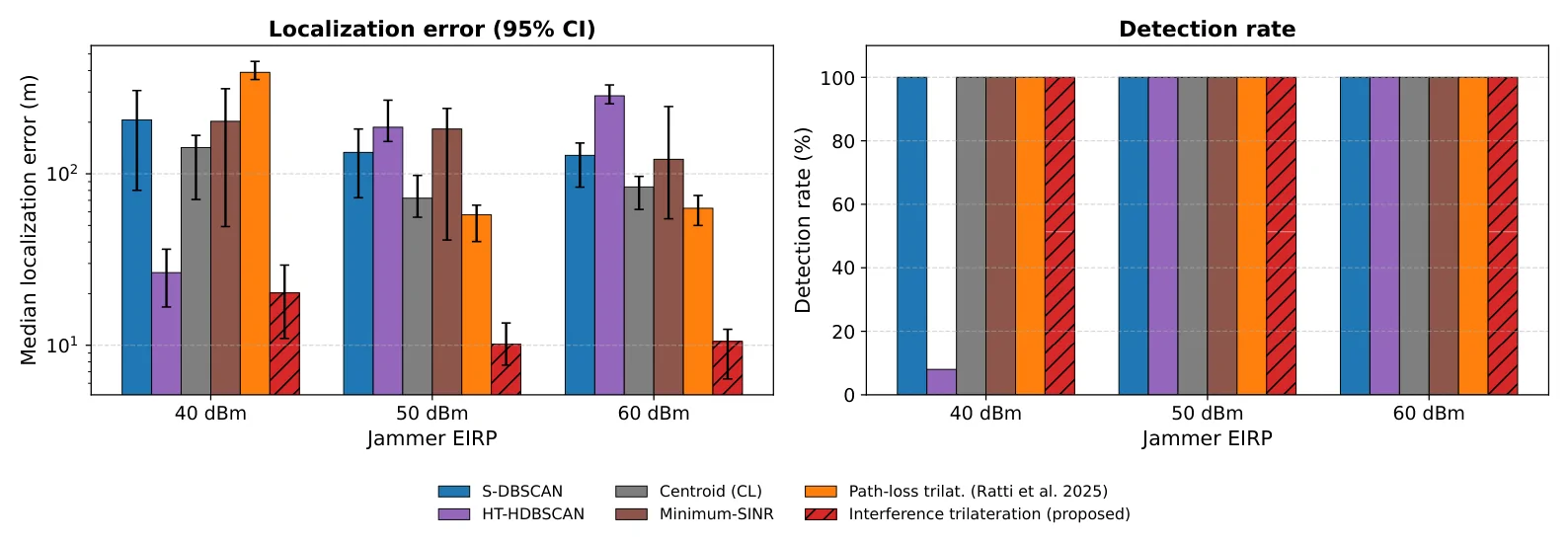
Scenario 2, 25 test realizations per jammer power: (**left**) median localization error of each method with its 95% bootstrap confidence interval (logarithmic scale); (**right**) detection rate. The HT-HDBSCAN bar at 40 dBm is computed over the two detected runs.

In the proposed framework, the clustering stages and the trilateration stage are complementary: S-DBSCAN provides a robust detection and sector identification after a few reports without the need for any RSRP measurement, while interference power trilateration provides the jammer coordinates after RSRP reports become available. Table 10 and Figure 14 report the same comparison for Scenario 2 (jammer between gNB 5 and gNB 2), in which every method uses the parameters tuned on Scenario 1. Again, no method raised a false alarm in the 25 jammer-free runs (0 of 1500 one-second windows). Because the jammer lies between two cells, S-DBSCAN, CL, and minimum-SINR detected all attacks, but their median errors remain 128–206 m, 72–142 m, and 122–202 m, respectively. HT-HDBSCAN detected only 2 of the 25 attacks at 40 dBm, and its error grew to 187 and 285 m at 50 and 60 dBm, i.e., when the victims cover most of the deployment. Path loss trilateration [26] reaches 58 and 63 m at 50 and 60 dBm, but 391 m at 40 dBm, again because the jammer EIRP is unknown. Interference power trilateration detected every attack, identified the correct sector in all runs, and attained median errors of 20, 10, and 11 m at 40, 50, and 60 dBm, i.e., six to seven times lower than the best other method that detected all attacks at each power (CL at 40 dBm and path loss trilateration at 50 and 60 dBm). With the correlated UE positioning error of 15 m per axis (Path B), these median errors become 19, 11, and 12 m, respectively. Because no parameter was re-tuned, these results also show that the proposed method generalizes to a different jammer position and a different number of UEs.

### 5.5. Latency and Scalability

O-RAN requires the control loops of xApps in the Near-RT RIC to complete within 1 s. The latency of the proposed framework has two components: the time needed to accumulate enough evidence of an attack, and the processing time of the algorithms.

**Evidence accumulation.** The E2 nodes report KPMs every $T_{E2}=50$ ms. With α=5 and $\delta_{\mathrm{score}}=15$, the stateful detector of S-DBSCAN declares a UE to be a victim after three consecutive degraded reports, i.e., 150 ms after its SINR has dropped. The stateless threshold of HT-HDBSCAN reacts within a single report (50 ms). Interference power trilateration averages the estimated jammer power over 1 s windows per UE, so it provides a first estimate about 1 s after the attack starts and refines it in the following windows. In the simulations, the median time to the first alarm was 1.0 s for interference power trilateration at all jammer powers. For S-DBSCAN, it was 0.6, 1.9, and 13.3 s in Scenario 1 and 0.4, 2.0, and 20.0 s in Scenario 2 at 60, 50, and 40 dBm, respectively, where interference power trilateration again raised the alarm after 1.0 s. A weak jammer degrades the SINR below $\gamma_{\mathrm{severe}}$ only for UEs that pass close to it, so the SINR-based detector must wait until at least two UEs have crossed this region. The interference power estimate instead reveals the jammer as soon as its power exceeds the noise floor by the INR threshold.

**Figure 15:**
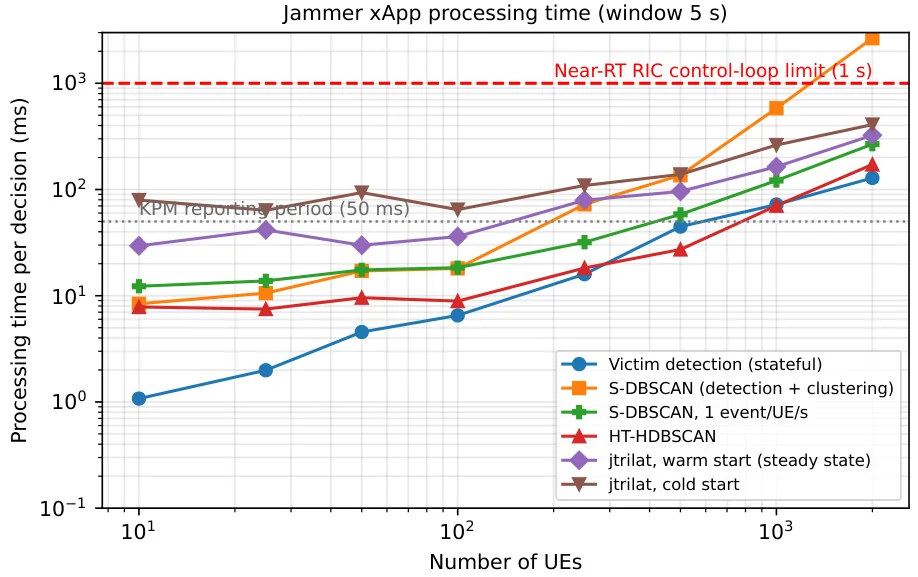
Processing time per decision of the Jammer xApp stages versus the number of UEs (5 s window, $T_{E2}=50$ ms). The dashed red line is the 1 s control-loop limit of the Near-RT RIC. Regular S-DBSCAN exceeds this limit with 2000 UEs (about 2.7 s), whereas the reduced version (one event per UE per second) takes about 270 ms. The values are processing times, and do not include E2 network delays.

**Processing time and scalability.** To assess the scalability with the number of UEs, we measured the processing time of each stage on a sliding window of 5 s of KPM reports (100 reports per UE at $T_{E2}=50$ ms) for UEs numbering from 10 to 2000, on an Intel Core Ultra 7 155H (single core). Figure 15 reports the results. Victim detection and HT-HDBSCAN grow linearly with the number of reports and remain below 175 ms even with 2000 UEs. The DBSCAN step of S-DBSCAN grows faster because its cost increases with the number of victim events, and reaches 2.7 s with 2000 UEs. Keeping the most degraded single event per UE per second bounds the number of points without affecting the estimate (localization errors of 98.5 and 101.6 m with 2000 UEs without and with this reduction) and keeps S-DBSCAN at 270 ms for 2000 UEs. Interference power trilateration takes 29–164 ms for up to 1000 UEs and 324 ms for 2000 UEs when initialized from the previous estimate, which is the steady-state operation of the xApp. The first estimate uses a coarse-to-fine multistart over the same 7 × 7 grid; the grid is screened with a closed-form cost and only the few most promising seeds are refined, so the cold-start trilateration calculation alone takes at most 0.41 s (2000 UEs). It is computed only once, when the attack is first detected. This value is not the duration of the full xApp loop, which also includes the other stages. In steady state, the four stages together take 0.15 s with 250 UEs and 0.89 s with 2000 UEs. These totals are obtained with S-DBSCAN restricted to one event per UE per second and with the trilateration initialized from the previous estimate; they fit within the 1 s budget of the Near-RT RIC for up to 2000 UEs per RIC on a single core only under these two conditions. Without the reduction, the DBSCAN step of S-DBSCAN alone takes 2.7 s with 2000 UEs and exceeds the 1 s limit (the total would be 3.3 s), so not every configuration meets the 1 s limit. Victim detection and HT-HDBSCAN together also fit within the 50 ms reporting period for up to 250 UEs. All the values are processing times of the algorithms; the full end-to-end latency, including the E2 network delays, was not measured.

## 6. Conclusions

Jamming threatens the physical layer of any wireless system, whether or not it follows the O-RAN architecture. What O-RAN adds is not a new vulnerability but a new means of defense: standardized access to RAN telemetry through the E2 interface and the possibility of deploying third-party applications in the RICs. In this paper, we exploit this opportunity using an O-RAN-native framework that detects jammers, identifies the affected sector, and localizes the jammer without the need for any specialized direction-finding hardware.

The proposed framework streams ${\mathrm{SINR}}_{L3}$ measurements from a KPM xApp to a Jammer xApp in the Near-RT RIC. The Jammer xApp identifies victim UEs with stateful SINR thresholds, detects the attack and the affected sector through spatial clustering (S-DBSCAN or HT-HDBSCAN), and localizes the jammer. We evaluated the framework on E2 KPM logs from ns-O-RAN using a downlink jamming model in which the jammer power adds to the noise in the SINR measured by the UEs. The evaluation comprised two scenarios, three jammer powers (40, 50, and 60 dBm EIRP), and a jammer-free control, each repeated over 30 independent Monte Carlo realizations, and applied every method to every scenario with the same parameters.

No false alarm was raised in the jammer-free runs. S-DBSCAN detected 80–100% of the attacks, depending on the jammer power, and identified the correct sector in 95–97% of the detected runs. However, SINR-based clustering alone localized the jammer only with median errors of 97–206 m (S-DBSCAN); at 50 and 60 dBm, this increased to 167–285 m (HT-HDBSCAN, which detected only 8–20% of the attacks at 40 dBm), no better than a simple centroid of the victim UEs. The SINR does not separate a strong jammer from a weak serving signal. Interference power trilateration, which combines SINR and RSRP, detected every attack within about 1 s and reduced the median localization error to 9–24 m in Scenario 1 and 10–20 m in Scenario 2, four to ten times lower than the baselines, with the same parameters in both scenarios. When the UE positions were not reported by the network, the SI-MAP estimator with a causal EKF estimated them in the RIC with a median error of 12.5–12.6 m in both scenarios (16.5 m under model mismatch); with a UE position error of this order, the median jammer localization error increases only to 11–26 m. In steady state, the processing time of all the stages of the Jammer xApp was 0.15 s per decision with 250 UEs and 0.89 s with 2000 UEs on a single CPU core, which is within the 1 s control-loop budget of the Near-RT RIC only for the reduced S-DBSCAN (one event per UE per second) with warm-start trilateration; regular S-DBSCAN takes about 2.7 s with 2000 UEs. It should be noted that these are processing times; the end-to-end latency including E2 delays was not measured. The results show that the clustering stages and trilateration stage are complementary: S-DBSCAN provides robust RSRP-free detection and sector identification, while interference power trilateration provides the jammer coordinates. The two clustering variants offer a trade-off: the stateful S-DBSCAN suppresses isolated SINR drops at the cost of keeping a small state per UE, while the stateless HT-HDBSCAN is simpler but relies on a stricter SINR threshold.

The present evaluation has some limitations. The jammer is modeled at the level of the L3 SINR measurements, not at the level of transport-block decoding. Inter-cell interference is not modeled, as in the ns-O-RAN SINR report. The UE ranging observables used for positioning are synthesized from the simulated geometry; with four closely spaced gNBs, the resulting UE-positioning accuracy (24 m at the 80th percentile) does not meet the 10 m 3GPP commercial target. The jammer is stationary. Path B is emulated by adding a correlated error to the ground-truth positions rather than by feeding the SI-MAP/EKF output end-to-end. Additionally, for SINR-only clustering methods, the density of victim events inherently reflects the dwell time of UEs in the degraded region rather than just the spatial extent of the attack. Furthermore, the current evaluation was performed offline using logged E2 KPM data; while the processing times of the reduced configuration fit within the Near-RT RIC budget, the end-to-end loop latency including E2 transport delays has not been measured in a live deployment.

Several directions for future work follow from these limitations. First, we plan to deploy the framework on physical O-RAN hardware with the FlexRIC platform in order to validate it under real channel conditions and with measured positioning observables. Second, we will assess its robustness to other jammer types (sweep, reactive, and protocol-aware jammers), to mobile jammers (which requires a sliding window or predictive localization stage), and to multi-jammer scenarios. Finally, in multi-vendor deployments where UE-level telemetry cannot be shared, the few parameters calibrated by the framework (detection thresholds, clustering parameters, and path loss exponent) could be exchanged between vendors through decentralized federated learning across O-RAN applications [38] as well as among participants without mutual trust [39], which would significantly reduce the need to share raw telemetry data. Hierarchical aggregation [40] and knowledge transfer between different models [41] could further reduce the communication cost and account for vendor-specific equipment.

## Figures and Tables

**Table 1:** Simulation parameters.

Parameter	Description	Value
*T*	Simulation duration	60.0 s
*L*	Scenario length (Bounds)	1000×1000 m
$N_{gNBs}$	Number of gNBs	4 (IDs: 2, 3, 4, 5)
$N_{UE}$	Number of UEs (Scenario 1/2)	10/15
$v_{UE}$	UE speed	[3.0–7.0] m/s
$P_{tx}$	gNB Tx power	44.0 dBm
$F_{c}$	Carrier frequency	3.5 GHz
*B*	Bandwidth	20 MHz
NF	UE noise figure	9 dB
$T_{E2}$	E2 KPM reporting period	50 ms
$Pos_{Jammer}$ (1)	Jammer pos (near gNB 3)	(710,510,1.5) m
$Pos_{Jammer}$ (2)	Jammer pos (between gNB 5 and gNB 2)	(450,413,1.5) m
$P_{Jammer}$	Jammer EIRP (isotropic)	40, 50, 60 dBm
$N_{MC}$	Monte Carlo realizations per configuration	30

**Table 2:** Framework hyperparameters (tuned on seeds 1–5).

Parameter	Description	Value
$\gamma_{\mathrm{detect}}$	Primary detection threshold	−5.0 dB
$\gamma_{\mathrm{severe}}$	Severe victim filter threshold	−12.0 dB
$\gamma_{th}$	Hard detection threshold (HT-HDBSCAN)	−20.0 dB
α	Stateful suspicion penalty	5
β	Stateful suspicion recovery	1
$\delta_{\mathrm{score}}$	Stateful victim declaration limit	15
ϵ	DBSCAN spatial search radius	400 m
$\mu_{pts}$	DBSCAN minimum cluster density	6
$\pi_{\mathrm{trim}}$	Outlier-trim percentile (S-DBSCAN)	75th
$m_{cluster}$	HDBSCAN minimum cluster size	3
$m_{samples}$	HDBSCAN minimum samples	2
${\mathrm{INR}}_{th}$	Interference-to-noise threshold (trilateration)	6 dB
*W*	Averaging window per UE (trilateration)	1 s
*n*	Path-loss exponent search range (trilateration)	[1.5,5]
$N_{\min}$	Minimum number of victim UEs for an alarm	2

**Table 3:** Two-dimensional UE localization error (m) pooled over the 25 test realizations of each scenario (Scenario 1: ten UEs, 150,000 snapshots; Scenario 2: fifteen UEs, 225,000 snapshots). Scenario 1 is evaluated with the observation model assumed by the estimator (matched) and with model mismatch.

Method	Median	p80	p90	RMSE
**Scenario 1, matched**				
SI-MAP snapshot	13.9	33.8	53.4	38.3
SI-MAP + causal EKF	**12.5**	**24.3**	**33.5**	**23.3**
Path-loss trilat. [26]	259.2	507.9	680.7	416.5
**Scenario 1, model mismatch**				
SI-MAP snapshot	20.4	49.3	78.4	56.5
SI-MAP + causal EKF	**16.5**	**32.4**	**45.7**	**34.4**
Path-loss trilat. [26]	259.2	507.9	680.7	416.5
**Scenario 2, matched**				
SI-MAP snapshot	14.0	33.8	54.3	38.6
SI-MAP + causal EKF	**12.6**	**24.2**	**33.7**	**23.6**
Path-loss trilat. [26]	259.8	512.0	679.2	413.8

**Table 4:** Per-UE mean absolute error (m) along the *x*- and *y*-axis for the SI-MAP snapshot estimator (Snap.) and the causal EKF (Trk.) (Scenario 1, seed 20). The average values are shown in bold.

UE ID	$x_{\mathrm{err}}$ Snap.	$y_{\mathrm{err}}$ Snap.	$x_{\mathrm{err}}$ Trk.	$y_{\mathrm{err}}$ Trk.
1	12.40	21.22	11.78	13.95
2	6.00	8.09	6.10	6.49
3	20.18	9.58	12.89	6.19
4	15.61	20.59	11.00	9.83
5	13.85	19.20	9.94	12.02
6	10.12	10.83	7.18	8.19
7	5.56	5.29	4.46	3.43
8	22.96	25.89	15.90	18.45
9	15.37	13.11	8.90	9.48
10	6.75	22.61	5.55	14.69
**Avg**	**12.88**	**15.64**	**9.37**	**10.27**

**Table 5:** UE classification in the realization of Figure 7 (Scenario 1, 50 dBm, seed 24).

UE ID	Final Classification	Min ${\mathrm{SINR}}_{L3}$ (dB)	Avg ${\mathrm{SINR}}_{L3}$ (dB)
**Jamming Victims**			
1	Victim	−31.0	3.2
5	Victim	−20.7	6.2
6	Victim	−33.2	2.0
7	Victim	−29.4	3.6
10	Victim	−18.2	7.3
**Legitimate UEs**			
2	Legitimate	−11.5	18.7
3	Legitimate	−13.4	14.7
4	Legitimate	−8.3	20.3
8	Legitimate	−9.5	18.8
9	Legitimate	−11.2	12.1

**Table 6:** Distances from the jammer position estimated by interference power trilateration to the gNBs (realization of Figure 7).

gNB ID	gNB Location	Distance (m)
2	(500.0,500.0)	204.0
3	(700.0,500.0)	5.3
4	(400.0,673.2)	351.6
5	(400.0,326.8)	348.2

**Table 7:** UE classification in the realization of Figure 10 (Scenario 2, 50 dBm, seed 17).

UE ID	Final Classification	Min ${\mathrm{SINR}}_{L3}$ (dB)	Avg ${\mathrm{SINR}}_{L3}$ (dB)
**Jamming Victims**			
1	Victim	−20.2	11.1
2	Victim	−20.2	6.6
3	Victim	−18.1	12.1
7	Victim	−14.2	11.8
8	Victim	−36.5	2.8
9	Victim	−41.8	−4.3
11	Victim	−19.6	12.8
13	Victim	−20.1	4.6
14	Victim	−25.9	5.7
15	Victim	−15.3	11.1
**Legitimate UEs**			
4	Legitimate	−9.8	12.9
5	Legitimate	−11.6	12.8
6	Legitimate	−8.9	10.2
10	Legitimate	−11.1	17.7
12	Legitimate	−16.3	23.7

**Table 8:** Distances from the jammer position to the gNBs estimated by interference power trilateration (realization of Figure 10).

gNB ID	gNB Location	Distance (m)
2	(500.0,500.0)	92.8
3	(700.0,500.0)	263.3
4	(400.0,673.2)	254.9
5	(400.0,326.8)	107.6

**Table 9:** Comparison with baseline methods in Scenario 1 (test seeds 6–30: 25 jammed runs per jammer power and 25 jammer-free runs). Det.: detection rate at 40 dBm (weakest jammer). FA: jammer-free runs with an alarm; no method raised an alarm in any of the 1500 one-second windows. Error: median localization error [m] with its 95% bootstrap confidence interval. Sector: fraction of detected runs, over all powers, in which the gNB closest to the estimate is gNB 3. The proposed method is shown in bold.

Method	Det. @40 dBm	FA	Error @40 dBm	Error @50 dBm	Error @60 dBm	Sector
Centroid (CL) [37]	84%	0/25	114 [92, 154]	87 [46, 109]	85 [64, 137]	87%
Minimum-SINR	84%	0/25	175 [135, 201]	158 [122, 204]	143 [113, 205]	93%
Path loss trilateration [26]	100%	0/25	295 [281, 339]	64 [43, 82]	110 [101, 134]	83%
HT-HDBSCAN	20%	0/25	96 [39, 132]	167 [150, 291]	226 [161, 349]	81%
S-DBSCAN	80%	0/25	148 [105, 190]	119 [95, 138]	97 [81, 122]	97%
**Interference trilateration (proposed)**	**100%**	**0/25**	**24 [17, 27]**	**15 [11, 17]**	**9 [4, 13]**	**100%**

**Table 10:** Comparison with baseline methods in Scenario 2 (test seeds 6–30: 25 jammed runs per jammer power and 25 jammer-free runs), with the parameters tuned on Scenario 1. Columns as in Table 9. Sector: fraction of detected runs, over all powers, in which the gNB closest to the estimate is gNB 5 or gNB 2. * Median over the two detected runs. The proposed method is shown in bold.

Method	Det. @40 dBm	FA	Error @40 dBm	Error @50 dBm	Error @60 dBm	Sector
Centroid (CL) [37]	100%	0/25	142 [71, 159]	72 [56, 98]	84 [62, 97]	99%
Minimum-SINR	100%	0/25	202 [49, 314]	183 [41, 241]	122 [55, 247]	88%
Path-loss trilateration [26]	100%	0/25	391 [355, 453]	58 [40, 66]	63 [50, 75]	88%
HT-HDBSCAN	8%	0/25	27 [17, 36] *	187 [154, 269]	285 [256, 330]	85%
S-DBSCAN	100%	0/25	206 [80, 313]	133 [73, 183]	128 [84, 151]	95%
**Interference trilateration (proposed)**	**100%**	**0/25**	**20 [11, 29]**	**10 [8, 13]**	**11 [6, 11]**	**100%**

## Data Availability

The data presented in this study are openly available on GitHub at https://github.com/steveratti/oran-jammer-detection-geolocation-dataset. The simulation dataset used in this revised version (30 independent runs per configuration for both scenarios) is provided as release v2.0 at https://github.com/steveratti/oran-jammer-detection-geolocation-dataset/releases/tag/v2.0, together with the ns-3 scenario and the modified ns-3 source files used to generate it (accessed on 12 October 2026).

## Abbreviations

The following abbreviations are used in this manuscript:

3GPP	Third-Generation Partnership Project
AI	Artificial Intelligence
AoA	Angle-of-Arrival
ARP	Address Resolution Protocol
B5G	Beyond 5G
BLER	Block Error Rate
CDF	Cumulative Distribution Function
CL	Centroid Localization
CNN	Convolutional Neural Network
CPU	Central Processing Unit
CRLB	Cramér–Rao Lower Bound
dApp	Distributed Application
DBSCAN	Density-Based Spatial Clustering of Applications with Noise
DDoS	Distributed Denial-of-Service
DoS	Denial-of-Service
E2SM	E2 Service Model
EIRP	Equivalent Isotropic Radiated Power
EKF	Extended Kalman Filter
EMG	Exponentially Modified Gaussian
FDRL	Federated Deep Reinforcement Learning
FR1	Frequency Range 1
gNB	Next-Generation NodeB
GPS	Global Positioning System
GT	Ground Truth
GUI	Graphical User Interface
HDBSCAN	Hierarchical DBSCAN
HT-HDBSCAN	Hard-Threshold HDBSCAN
IDS	Intrusion Detection System
INR	Interference-to-Noise Ratio
IoT	Internet of Things
IQ	In-phase and Quadrature
J/S	Jamming-to-Signal
KPI	Key Performance Indicator
KPM	Key Performance Measurement
L3	Layer 3
LOS	Line-of-Sight
mmWave	Millimeter-Wave
MCS	Modulation and Coding Scheme
MIMO	Multiple-Input Multiple-Output
ML	Machine Learning
MRD	Mutual Reachability Distance
Near-RT RIC	Near-Real-Time RAN Intelligent Controller
NF	Noise Figure
NLOS	Non-Line-of-Sight
Non-RT RIC	Non-Real-Time RAN Intelligent Controller
O-CU	O-RAN Centralized Unit
O-DU	O-RAN Distributed Unit
O-RAN	Open Radio Access Network
O-RU	O-RAN Radio Unit
OSS	Operations Support System
PHY	Physical-Layer
PL	Path Loss
PRS	Positioning Reference Signal
QoS	Quality of Service
RAN	Radio Access Network
rApp	Non-RT RIC application
RF	Radio Frequency
RIC	RAN Intelligent Controller
RMS	Root Mean Square
RMSE	Root Mean Square Error
RRC	Radio Resource Control
RSRP	Reference Signal Received Power
S-DBSCAN	Stateful DBSCAN
SAJD	Self-Adaptive Jammer Detection
SDR	Software-Defined Radio
SI-MAP	SINR-Informed Maximum A Posteriori
SINR	Signal-to-Interference-plus-Noise Ratio
SMO	Service Management and Orchestration
SVM	Support Vector Machine
UE	User Equipment
UMi	Urban Micro
xApp	Near-RT RIC application

## References

[1] Lichtman M., Rao R., Marojevic V., Reed J., Jover R.P. (2018). 5G NR jamming, spoofing, and sniffing: Threat assessment and mitigation. Proceedings of the 2018 IEEE International Conference on Communications Workshops (ICC Workshops).

[2] Azariah W., Bimo F.A., Lin C.W., Cheng R.G., Nikaein N., Jana R. (2024). A survey on open radio access networks: Challenges, research directions, and open source approaches. Sensors.

[3] Lacava A., Polese M., Sivaraj R., Soundrarajan R., Bhati B.S., Singh T., Zugno T., Cuomo F., Melodia T. (2023). Programmable and customized intelligence for traffic steering in 5G networks using open RAN architectures. IEEE Trans. Mob. Comput..

[4] Arnaz A., Lipman J., Abolhasan M., Hiltunen M. (2022). Toward integrating intelligence and programmability in open radio access networks: A comprehensive survey. IEEE Access.

[5] Polese M., Bonati L., D’oro S., Basagni S., Melodia T. (2023). Understanding O-RAN: Architecture, interfaces, algorithms, security, and research challenges. IEEE Commun. Surv. Tutor..

[6] Bonati L., D’Oro S., Polese M., Basagni S., Melodia T. (2021). Intelligence and learning in O-RAN for data-driven NextG cellular networks. IEEE Commun. Mag..

[7] Elyasi A., Ashdown A., Rumman K.M., Restuccia F. (2025). O-RAN xApps: Survey and Research Challenges. SSRN Preprint.

[8] Ahmad I., Kumar T., Liyanage M., Okwuibe J., Ylianttila M., Gurtov A. (2018). Overview of 5G security challenges and solutions. IEEE Commun. Stand. Mag..

[9] Amachaghi E.N., Shojafar M., Foh C.H., Moessner K. (2024). A survey for intrusion detection systems in open RAN. IEEE Access.

[10] Balogun H.O., Adanigbo O.S. (2024). Implementing Cyber Threat Intelligence and Monitoring in 5G O-RAN: Proactive Protection Against Evolving Threats. Iconic Res. Eng. J..

[11] Tan H., Li Z., Xie N., Lu J., Niyato D. (2023). Detection of jamming attacks for the physical-layer authentication. IEEE Trans. Wirel. Commun..

[12] Hong S., Kim K., Lee S.H. (2023). A hybrid jamming detection algorithm for wireless communications: Simultaneous classification of known attacks and detection of unknown attacks. IEEE Commun. Lett..

[13] Azkaei B., Joshi K.C., Exarchakos G. (2025). Machine Learning-Driven Anomaly Detection for 5G O-RAN Performance Metrics. Proceedings of the IEEE INFOCOM 2025-IEEE Conference on Computer Communications Workshops (INFOCOM WKSHPS).

[14] Alves P.V., Goldbarg M.A., Barros W.K., Rego I.D., JMT Filho V., Martins A.M., de Sousa V.A., Fontes R.d.R., Aranha E.H.d.S., Neto A.V. (2023). Machine learning applied to anomaly detection on 5g o-ran architecture. Procedia Comput. Sci..

[15] Xavier B.M., Dzaferagic M., Vilà I., Martinello M., Ruffini M. (2024). Cross-domain AI for early attack detection and defense against malicious flows in O-RAN. Proceedings of the ICC 2024-IEEE International Conference on Communications.

[16] Hussain A., Abughanam N., Qadir J., Mohamed A. (2022). Jamming detection in iot wireless networks: An edge-ai based approach. Proceedings of the 12th International Conference on the Internet of Things.

[17] Hanegraaf M., Sciancalepore S., Oligeri G. (2025). Weak-Jamming Detection in IEEE 802.11 Networks: Techniques, Scenarios and Mobility. arXiv.

[18] Porambage P., Christopoulou M., Han B., Habibi M.A., Bogucka H., Kryszkiewicz P. (2024). Security, privacy, and trust for open radio access networks in 6G. IEEE Open J. Commun. Soc..

[19] Aizikovich E., Mimran D., Grolman E., Elovici Y., Shabtai A. (2025). Rogue Cell: Adversarial Attack and Defense in Untrusted O-RAN Setup Exploiting the Traffic Steering xApp. arXiv.

[20] Soleymani S.A., Eslamnejad M., Alimohammadi H., Akbas A., Foh C.H., Shojafar M. (2024). DDoS Detection and Mitigation Using d/xApp in O-RAN. Proceedings of the 2024 IEEE Future Networks World Forum (FNWF).

[21] Habibur Rahman M., Hossen M.S., Stephenson N.H., Shah V.K., Da Silva A. (2025). A Demonstration of Self-Adaptive Jamming Attack Detection in AI/ML Integrated O-RAN. arXiv.

[22] Abou El Houda Z., Moudoud H., Brik B. (2024). Federated deep reinforcement learning for efficient jamming attack mitigation in O-RAN. IEEE Trans. Veh. Technol..

[23] Hoffmann M., Kulacz L., Baldo O., Pakula M., Raghothaman B. (2026). Jamming Detection in 5G/6G Networks: From O-RAN Concept to OCUDU Deployment. arXiv.

[24] Nagampally R.R. (2025). Interference Avoidance and Mitigation in 5G and Beyond Networks. Ph.D. Thesis.

[25] Bartoletti S., Conti A., Dardari D., Giorgetti A. (2018). 5G Localization and Context-Awareness.

[26] Ratti S.A., Tuzi A., Piccioni A., Marotta A., Cassioli D., Graziosi F. (2025). Exploiting O-RAN as an Effective Solution for Malicious User Localization. Proceedings of the 2025 17th International Conference on COMmunication Systems and NETworks (COMSNETS).

[27] Radivilova T., Kirichenko L., Lemeshko O., Ageyev D., Mulesa O., Ilkov A. (2021). Analysis of anomaly detection and identification methods in 5G traffic. Proceedings of the 2021 11th IEEE International Conference on Intelligent Data Acquisition and Advanced Computing Systems: Technology and Applications (IDAACS).

[28] Ester M., Kriegel H.P., Sander J., Xu X. (1996). A Density-Based Algorithm for Discovering Clusters in Large Spatial Databases with Noise. Proceedings of the 2nd International Conference on Knowledge Discovery and Data Mining (KDD).

[29] Campello R.J.G.B., Moulavi D., Sander J. (2013). Density-Based Clustering Based on Hierarchical Density Estimates. Proceedings of the 17th Pacific-Asia Conference on Knowledge Discovery and Data Mining (PAKDD).

[30] Bout E., Brighente A., Conti M., Loscrì V. (2022). FOLPETTI: A novel multi-armed bandit smart attack for wireless networks. Proceedings of the 17th International Conference on Availability, Reliability and Security.

[31] O-RAN Alliance (2023). O-RAN E2 Service Model (E2SM) KPM IWF Technical Specification.

[32] Lacava A., Bordin M., Polese M., Sivaraj R., Zugno T., Cuomo F., Melodia T. (2023). ns-o-ran: Simulating o-ran 5g systems in ns-3. Proceedings of the 2023 Workshop on ns-3.

[33] Polese M., Bonati L., D’Oro S., Johari P., Villa D., Velumani S., Gangula R., Tsampazi M., Robinson C.P., Gemmi G. (2024). Colosseum: The open RAN digital twin. IEEE Open J. Commun. Soc..

[34] Xavier B.M., Dzaferagic M., Martinello M., Ruffini M. (2024). Performance measurement dataset for open RAN with user mobility and security threats. Comput. Netw..

[35] Pereira C., Aguiar A. (2013). A realistic RF jamming model for vehicular networks: Design and validation. Proceedings of the 2013 IEEE 24th Annual International Symposium on Personal, Indoor, and Mobile Radio Communications (PIMRC).

[36] 3rd Generation Partnership Project (3GPP) (2020). Study on Channel Model for Frequencies from 0.5 to 100 GHz.

[37] Liu H., Liu Z., Chen Y., Xu W. (2011). Determining the Position of a Jammer Using a Virtual-Force Iterative Approach. Wirel. Netw..

[38] Spantideas S.T., Giannopoulos A.E., Levis G.A., Trakadas P. (2026). FedRA: A Fully Decentralized Federated Learning Framework for Robust Intelligence Sharing across O-RAN dApps. IEEE Commun. Mag..

[39] Qin Z., Yan X., Zhou M., Deng S. (2024). BlockDFL: A Blockchain-based Fully Decentralized Peer-to-Peer Federated Learning Framework. Proceedings of the ACM Web Conference 2024 (WWW ’24), 13–17 May 2024, Singapore.

[40] Deng Y., Lyu F., Xia T., Zhou Y., Zhang Y., Ren J., Yang Y. (2024). A Communication-Efficient Hierarchical Federated Learning Framework via Shaping Data Distribution at Edge. IEEE/ACM Trans. Netw..

[41] Deng Y., Ren J., Tang C., Lyu F., Liu Y., Zhang Y. (2023). A Hierarchical Knowledge Transfer Framework for Heterogeneous Federated Learning. Proceedings of the IEEE INFOCOM 2023—IEEE Conference on Computer Communications.

